# An Alternative Approach to ChIP-Seq Normalization Enables Detection of Genome-Wide Changes in Histone H3 Lysine 27 Trimethylation upon EZH2 Inhibition

**DOI:** 10.1371/journal.pone.0166438

**Published:** 2016-11-22

**Authors:** Brian Egan, Chih-Chi Yuan, Madeleine Lisa Craske, Paul Labhart, Gulfem D. Guler, David Arnott, Tobias M. Maile, Jennifer Busby, Chisato Henry, Theresa K. Kelly, Charles A. Tindell, Suchit Jhunjhunwala, Feng Zhao, Charlie Hatton, Barbara M. Bryant, Marie Classon, Patrick Trojer

**Affiliations:** 1 Active Motif Inc., Carlsbad, California, United States of America; 2 Constellation Pharmaceuticals Inc., Cambridge, Massachusetts, United States of America; 3 Department of Molecular Oncology, Genentech Inc., South San Francisco, California, United States of America; 4 Department of Protein Chemistry, Genentech Inc., South San Francisco, California, United States of America; 5 Department of Bioinformatics, Genentech Inc., South San Francisco, California, United States of America; Emory University Rollins School of Public Health, UNITED STATES

## Abstract

Chromatin immunoprecipitation and DNA sequencing (ChIP-seq) has been instrumental in inferring the roles of histone post-translational modifications in the regulation of transcription, chromatin compaction and other cellular processes that require modulation of chromatin structure. However, analysis of ChIP-seq data is challenging when the manipulation of a chromatin-modifying enzyme significantly affects global levels of histone post-translational modifications. For example, small molecule inhibition of the methyltransferase EZH2 reduces global levels of histone H3 lysine 27 trimethylation (H3K27me3). However, standard ChIP-seq normalization and analysis methods fail to detect a decrease upon EZH2 inhibitor treatment. We overcome this challenge by employing an alternative normalization approach that is based on the addition of *Drosophila melanogaster* chromatin and a *D*. *melanogaster-*specific antibody into standard ChIP reactions. Specifically, the use of an antibody that exclusively recognizes the *D*. *melanogaster* histone variant H2Av enables precipitation of *D*. *melanogaster* chromatin as a minor fraction of the total ChIP DNA. The *D*. *melanogaster* ChIP-seq tags are used to normalize the human ChIP-seq data from DMSO and EZH2 inhibitor-treated samples. Employing this strategy, a substantial reduction in H3K27me3 signal is now observed in ChIP-seq data from EZH2 inhibitor treated samples.

## Introduction

ChIP-seq is a powerful and commonly used technique for the detection of transcription factor binding patterns and histone post-translational modification (PTM) occupancy profiles across the entire genome [1]. ChIP-seq data in many different cell types and contexts have been used to generate genome-wide chromatin modification maps that have provided significant insight into the general relationship between transcriptomic and epigenomic landscapes [2, 3]. These cell type comparisons have revealed substantial lineage-related differences in the profiles of specific histone PTMs across genomes. However, manipulation of a given biological context, such as comparisons of knockdown or knockout of individual histone modifying enzymes or their respective inhibition with small molecules, may potentially involve subtle alterations to the PTM landscape rather than resulting in a completely different pattern. Therefore, in recent years, more complex statistical methods, software programs and computational models have been developed in an attempt to adequately compare ChIP-seq data sets and reliably reveal the differences [4–7].

Identifying differences between data sets becomes more challenging when differences are not just occurring at specific sites across the genome, but involves global modification changes. An example would be a setting where an increase or decrease of a particular histone PTM occurs at all or most occupied sites across the genome, as is frequently the case when studying the effects of chromatin modifying enzyme inhibitors. Impairing the function of a histone methyltransferase (HMT) can result in a reduction in bulk methylation levels at the targeted histone residue, which in the case of H3K27 methylation affects a large part of the genome. In these instances, currently available bioinformatic-based normalization methods are not applicable since they assume invariance in the signal to noise ratio, the background signal level, or the height of shared peaks.

Several recently described approaches alter the ChIP protocol by adding material that can be used to normalize the signal. For example, ChIP-Rx is based on the addition of a constant amount of reference cells from a different species, and allows for the genome-wide quantitative comparison of histone modifications across different biological samples [8]. The method ultimately depends on the ability of the experimental ChIP antibody to recognize the histone modification of interest in both the reference and experimental species. Precipitated reference DNA is sequenced along with precipitated experimental DNA, and thus reference sequence reads provide a means to normalize across biological samples. This approach was successfully used to visualize genome-wide changes in H3K79me2 levels upon treatment with a small molecule inhibitor of DOT1L, the sole H3K79-specific HMT [8].

EZH2 is the major H3K27-specific HMT with an important role in transcriptional repression. Genomic and transcriptomic data have identified EZH2 as a candidate oncology target in a number of human malignancies, including prostate, breast and hematological malignancies [9, 10]. Recurrent, somatic mutations in EZH2, which alter its substrate specificity and increase global H3K27me3 levels, have been found in diffuse large B-cell lymphoma, follicular lymphoma and melanoma [11–17]. Small molecule inhibitors of EZH2 have recently been discovered [18–24], and a number of these compounds are currently being clinically developed as a promising therapeutic for the treatment of cancer. In order to better understand the molecular events impacted by EZH2 inhibition in human cancer cells, it is important to monitor inhibitor induced alterations in H3K27me3 patterns across the genome. However, detection of EZH2 inhibitor-induced global differences in H3K27me3 levels by standard ChIP-seq protocols and data analysis methods has proven difficult. Therefore, we developed an approach that allows for the detection of global and locus-specific changes in H3K27me3 levels in human cell models through the use of a second antibody added to each ChIP reaction. Similar to previously published methods, [8, 25] we use a chromatin spike-in approach, however, while these methods depend on the ability of the experimental antibody to recognize chromatin features on spiked-in chromatin from a second species, we sought to establish a method that is independent of the cross-reactivity potential of the experimental antibody. Importantly, an antibody exclusively recognizing the *D*. *melanogaster*-specific histone variant H2Av is added to all ChIP samples along with a constant amount of *D*. *melanogaster* chromatin. We employed this strategy effectively in two different cancer cell contexts using two different EZH2 inhibitors. Normalization using spike-in standards in these cell systems effectively uncover the genome-wide reduction in H3K27me3 occupancy upon inhibitor treatment, while other histone methylation marks not affected by EZH2 inhibitors were unaffected by the normalization. We suggest that this approach provides a broadly applicable solution for the normalization of ChIP-seq data sets.

## Materials and Methods

### Cells and Treatments

PC9, a lung derived human adenocarcinoma cell line, was kindly provided by Dr. Kazuto Nishio (National Cancer Center Hospital, Tokyo, Japan) and cultured at 37°C with 5% CO_2_ in RPMI-1640 (Invitrogen) supplemented with 10% FBS (HyClone) and antibiotics (Invitrogen). Cells were treated with 1 μM EZH2 inhibitor GSK126 in the same media with 5% FBS for 5 days. KARPAS-422, a human B cell lymphoma cell line containing a mono-allelic activating mutation in EZH2, was obtained from the Leibniz Institute DSMZ German Collection of Microorganisms and Cell Cultures, and cultured at 37°C with 5% CO_2_ in RPMI-1640 medium (Invitrogen) supplemented with 10% FBS (HyClone) and antibiotics (Invitrogen). Cells were treated with 1.5 μM CPI-360 for 4 and 8 days. Schneider (S2), a *D*. *melanogaster* epithelial cell line, was obtained from ATCC and cultured at 28°C without CO_2_ in Schneider’s medium (04-351Q, Lonza) containing 1% FBS. OSS, a *D*. *melanogaster* ovarian somatic sheath cell line, was obtained from the Drosophila Genomics Resource Center and cultured as described [26]. Immunoprecipitation and western blotting from cell lysates and histone mass spectrometric analyses are described in the supporting information.

### ChIP

PC9, KARPAS-422, OSS, and S2 cells were fixed with 1% formaldehyde (Sigma) for 15 min and quenched with 0.125 M glycine. Cells were washed twice in PBS and three times in cell lysis buffer (10 mM Tris-HCl pH 7.5, 10 mM NaCl, 3 mM MgCl_2_, 0.5% NP-40). KARPAS-422 cell pellet then was re-suspended in miccrococcal nuclease (MNase) reaction buffer (10 mM Tris-HCl pH 8, 10 mM NaCl, 3 mM MgCl_2_, 1 mM CaCl_2_, 4% NP-40) plus 20000 U of MNase (NEB M0247S) at 37°C for 15 min. Digestion was terminated by the addition of EGTA to 3 mM, SDS concentration was increased to 1%, and NaCl concentration was increased to 150 mM. Chromatin was sonicated at 15% output for 6 x 10 sec (Sonic Dismembrator 500, Fisher Scientific). Cell debris was removed by centrifugation at 20,000 x g for 10 min, and the resulting chromatin was diluted with IP dilution buffer (20 mM Tris-HCl pH 8, 2 mM EDTA, 1% Triton X-100, 150 mM NaCl) and pre-cleared with protein G-coupled magnetic beads (Invitrogen). PC9, OSS, and S2 cell pellets were treated the same as KARPAS-422 cells except the cells were sonicated at 15% output for 30 x 10 sec (EpiShear probe sonicator, Active Motif #53051) in sonication buffer (10 mM Tris-HCl pH 8, 150 mM NaCl, 3 mM MgCl_2_, 1 mM CaCl_2_, 4% NP-40, 1% SDS).

For antibody titration Experiments in S4 Fig, 12 μg of KARPAS-422 chromatin was incubated with 250 ng to 8 μg of H3K27me3 antibody (Cell Signaling, #9733), rabbit IgG (Jackson ImmunoResearch Lab 011-000-003), or 200 ng to 6.4 μg of a rabbit polyclonal antibody raised against the C-terminus of *D*. *melanogaster* H2Av (Active Motif #39715) overnight at 4°C. For titration with *D*. *melanogaster* chromatin, 2.85 μg of S2 chromatin was incubated with 125 ng to 4 μg of H3K27me3 antibody (Cell Signaling, #9733), rabbit IgG (Jackson ImmunoResearch Lab 011-000-003), or 200 ng to 6.4 μg of H2Av antibody (Active Motif #39715) overnight at 4°C. Antibody-chromatin complex was captured by the addition of protein G magnetic beads and the beads were washed once with IP dilution buffer, twice with wash buffer 1 (20 mM Tris-HCl pH 8, 2 mM EDTA, 1% Triton X-100, 0.1% SDS, 150 mM NaCl), once with wash buffer 2 (20 mM Tris-HCl pH 8, 2 mM EDTA, 1% Triton X-100, 0.1% SDS, 500 mM NaCl), once with wash buffer 3 (10 mM Tris-HCl pH 8, 1 mM EDTA, 250 mM LiCl, 1% NP-40, 1% deoxycholate), and once with TE (10 mM Tris-HCl pH 8, 1 mM EDTA). DNA-protein complex was eluted twice with 100 μl elution buffer (25 mM Tris-HCl pH 7.5, 10 mM EDTA, 0.5% SDS) at 65°C for 15 min with shaking at 1200 rpm (Eppendorf ThermoMixer C). Eluted chromatin was treated with 10 mg of RNAse A at 37°C for 1 h followed by proteinase K at 50°C for 2 h. Samples were then de-crosslinked at 65°C for 5 h. Reverse crosslinked DNA was purified by a PCR purification column (Qiaquick PCR purification, Qiagen 28106), and eluted with 150 μl of buffer EB (10 mM Tris-HCl pH 8) for qPCR. Small aliquots of input chromatin samples were reverse crosslinked, RNase A, proteinase K digested and purified with PCR purification columns as described above. The concentration of the purified DNA was measured with Qubit Fluorometric Quantitation (Invitrogen) as a reference for the amount of chromatin used in each ChIP reaction. Quantitative polymerase chain reaction analysis of purified ChIP DNA (ChIP-qPCR) is described in the supporting information.

### ChIP Sequencing and Data Analysis

For ChIP-seq experiments, 30 μg of chromatin was pre-cleared with protein A agarose beads (Invitrogen). Genomic DNA regions of interest were isolated using 3 μg of H3K27me3 antibody (Cell Signaling, #9733), 4 μg anti-H3K4me3, a rabbit monoclonal antibody raised against a synthetic peptide corresponding to the N-terminus of histone H3 and containing a tri-methylated lysine 4 (Cell Signaling, #9751) or 5 μl of anti-H3K9me3 serum from a rabbit immunized with a synthetic peptide corresponding to the N-terminus of histone H3 and containing a tri-methylated lysine 9 (Active Motif, #39161). ChIP reactions had a volume of 250 μl and were processed as described above. For ChIP-seq experiments with spike-in *D*. *melanogaster* chromatin, the samples were prepared and processed as above but with the addition of 0.4 μg of H2Av antibody (Active Motif, #39715) and 750 ng of *D*. *melanogaster* S2 cell chromatin for KARPAS-422 replicate #1 and 3.75 μg for replicate #2 and #3 (to increase the number of *D*. *melanogaster* tags) or 750 ng of *D*. *melanogaster* OSS cell chromatin for PC9 experiments.

ChIP and input DNA libraries were prepared for Illumina sequencing by using one half of the total ChIP DNA from each reaction. The amount of ChIP DNA was not determined for all replicates but known quantities are presented in S1 Table. Libraries were prepared using the standard consecutive enzymatic steps of end-polishing, dA-addition, and adaptor ligation using Active Motif’s custom liquid handling robotics pipeline. The adaptor-ligated libraries were purified using Agencourt AMPure XP beads and amplified with barcoded primers for 15 cycles. The resulting libraries were purified with Agencourt AMPure XP beads and quantified to assess quality of the amplification reactions. Amplified DNAs were sequenced in multiplex on an Illumina HiSeq 2500 using 50 bp single end sequencing. Coverage ranged from 25 million to 40 million tags per ChIP-seq sample.

Sequences (50-nt tags, single end) were separately aligned to either the human genome (hg19) or the *D*. *melanogaster melanogaster* genome (dm3) using the BWA algorithm (default settings). Duplicate tags were removed and only uniquely mapped tags (mapping quality > 25) were used for further analysis. Alignments were extended *in silico* at their 3’-ends to a length of 200 bp, which is the average genomic fragment length of the library molecules, and assigned to 32-nt bins along the genome. Peak locations were identified using the SICER algorithm (v1.1) with an E-value of 1 based on random background (SICER-rb; without control library file). The gap size parameter was set at 600 bp for H3K9me3 and H3K27me3, and 200 bp for H3K4me3. Peak regions were defined to be the union of peak intervals in control and treated samples, and average signal was reported for each region. Our standard normalization for the comparison of multiple samples was implemented by normalizing human tag numbers in each sample to the same number of tags present in the sample with the lowest number of uniquely mapped tags. Tag removal was performed by randomly removing excess tags from the larger files. The spike-in normalization was performed by counting *D*. *melanogaster* tags in each sample and using those tag counts to generate correction factors; calculated as DMSO tags/inhibitor tags. The primary method of mapping *D*. *melanogaster* tags used a partial dm3 reference genome containing only H2Av bound regions that we identified in H2Av ChIP-seq experiments using only *D*. *melanogaster* chromatin from S2 or OSS cells. The alternate method of mapping *D*. *melanogaster* tags used the entire dm3 reference genome. Correction factors were then applied to the human tag counts for all samples and scaling of human tags was performed by randomly removing tags as dictated by the scaling factor.

The Integrated Genomics Viewer (IGV) v2.3 (https://www.broadinstitute.org/igv/) was used for visualization of ChIP-seq data sets.

## Results

### Standard ChIP-Seq Protocols Fail to Reveal EZH2 Inhibitor Mediated Global Decreases in H3K27me3

**Fig 1:**
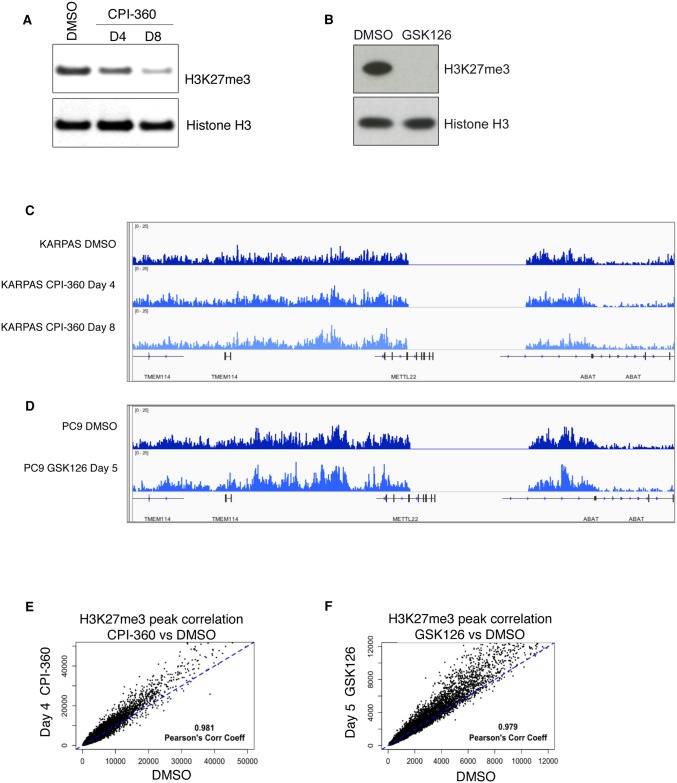
EZH2 inhibition reduces global H3K27me3 levels, however standard ChIP-seq methods do not reveal the reduction. **(A)** Western blot showing reduced global H3K27me3 levels in KARPAS-422 cells treated with 1.5 μM CPI-360 for 4 and 8 days. Whole cell extracts were resolved by SDS page and immuno-blotted with anti-H3K27me3. Anti-H3 immuno-blots show equal levels of total H3. **(B)** Western blot showing reduced global H3K27me3 levels in PC9 cells treated with 1 μM of GSK126 for 5 days. Whole cell extracts were resolved by SDS page and immuno-blotted with anti-H3K27me3. Anti-H3 immuno-blots show equal levels of total H3. **(C, D)** Representation of H3K27me3 ChIP-seq data using IGV. No obvious differences are detected in CPI-360 (C) and GSK126 (D) treated KARPAS-422 and PC9 cells when compared to vehicle-treated controls. **(E, F)** Genome-wide data from H3K27me3 ChIP-seq experiments under different treatment conditions are represented as scatter plots.

To study the impact of EZH2 inhibition on the genome-wide distribution of H3K27me3, we treated two different human cancer cell lines, KARPAS-422 germinal center B-cell like diffuse large B-cell lymphoma and PC9 lung adenocarcinoma, with different EZH2 inhibitors, CPI-360 [18] and GSK126 [23]. Using western blots we detected a substantial reduction in total H3K27me3 levels in the presence of the inhibitors in both cellular contexts (Fig 1A and 1B). Mass spectrometry analysis using the same samples confirmed the global decrease in H3K27me3 abundance (S1 Fig). Consistent with previous reports [18], global levels of other histone methylation marks, such as H3K4me3 or H3K9me3, were unaffected by the inhibitor treatment (S1 Fig).

To better understand how EZH2 inhibitor-mediated reduction in H3K27me3 levels affects the distribution of this modification across the genome, we performed H3K27me3 ChIP-seq in cells treated with EZH2 inhibitors as compared to untreated cells. Data was obtained from multiple biological replicates from each of the two cell models and analyzed using standard sequencing depth normalization. Despite good signal reproducibility across replicates (S1 Fig), these ChIP-seq studies failed to show locus specific (Fig 1C and 1D) or general (Fig 1E and 1F) decreases in H3K27me3 occupancy in the EZH2 inhibitor-treated samples when compared to DMSO-treated controls.

### ChIP-qPCR Detects Locus-Specific H3K27me3 Reduction upon EZH2 Inhibitor Treatment

**Fig 2:**
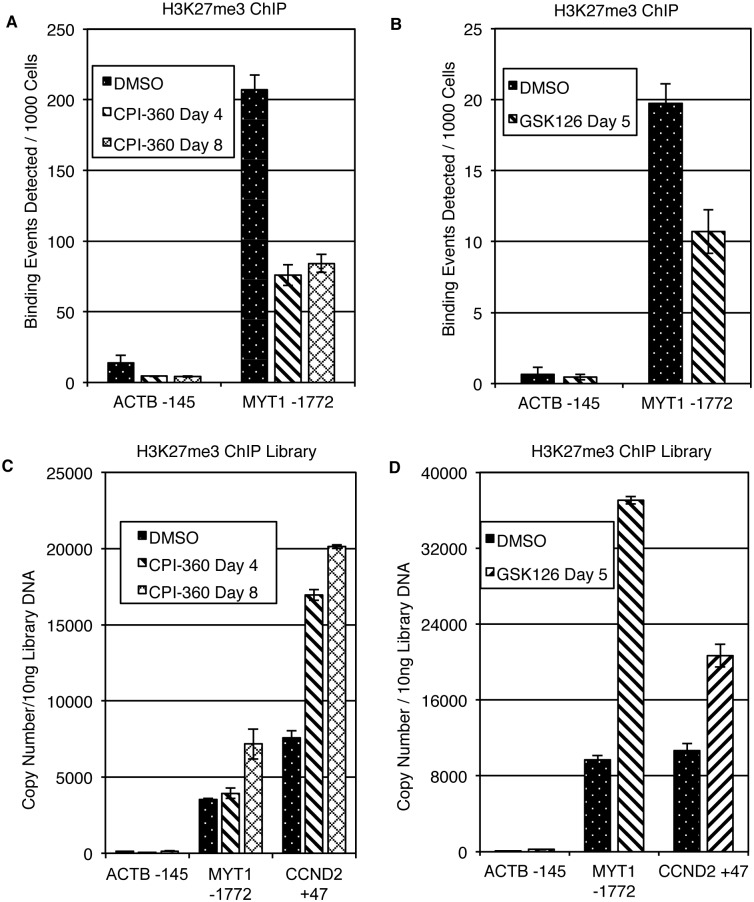
Reduced H3K27me3 binding is detected by ChIP-qPCR. **(A)** ChIP was performed using chromatin from KARPAS-422 cells treated with the EZH2 inhibitor CPI-360. qPCR using the positive control primer *MYT1* showed reduced H3K27me3 occupancy in the presence of the inhibitor. **(B)** ChIP was performed using chromatin from PC9 cells treated with the EZH2 inhibitor GSK126. qPCR using the positive control primer *MYT1* showed reduced H3K27me3 occupancy in cells treated with the inhibitor. (**C**) Libraries were generated from KARPAS-422 cells using 15 cycles of PCR amplification. Library DNA was diluted and qPCR was performed using positive control primers for *MYT1* and *CCND2*. (**D**) Libraries were generated from PC9 cells as described in (C) and library DNA was used for qPCR using positive control primers for *MYT1* and *CCND2*. All experiments are represented as the mean of two independent experiments with qPCRs performed in triplicate ±SD. The *ACTB* promoter served as a negative control for all experiments.

Changes in H3K27me3 at specific loci upon EZH2 inhibitor treatment have been observed previously [18, 27]. To confirm that H3K27me3 loss at specific genomic loci is detectable under the employed ChIP conditions, H3K27me3 ChIP using chromatin from cells treated with DMSO or EZH2 inhibitors GSK126 and CPI-360 were performed, followed by qPCR to explore H3K27me3 enrichment at a well-characterized PRC2 target gene, *MYT1*. As expected, H3K27me3 enrichment was reduced in the *MYT1* promoter region in the presence of both EZH2 inhibitors (Fig 2A and 2B). Compound specificity toward H3K27me3 was underscored by the lack of gene-specific changes in H3K4me3 and H3K9me3 enrichment (S2 Fig). These results indicate that the employed ChIP procedure is adequate to detect changes in H3K27me3 occupancy by qPCR.

However, qPCRs from amplified ChIP-libraries before sequencing showed that the differences between DMSO and EZH2 inhibitor treated samples were no longer present (Fig 2C and 2D). In fact, there was a trend towards an increased signal in EZH2 inhibitor treated samples despite the fact that (1) the precipitated total DNA amounts were reduced in compound treated samples (S1 Table) and (2) library yields were similar across untreated and treated samples (S2 Table). These experiments show that standard library generation protocols, in addition to subsequent bioinformatics analysis, may be suboptimal for accurate quantitative representation of global histone PTM changes in biological samples from different treatment conditions. Others [28, 8] were confronted with a similar problem and used a synthetic RNA spike-in standard or *D*. *melanogaster* chromatin to normalize global RNA sequencing and ChIP-seq profiling data, respectively.

### Designing a Spike-In Normalization Strategy

An appropriate spike-in standard for ChIP-seq will serve as a reference for sample comparison and normalization and should accomplish the following: 1) Spike-in material is present in all samples in equal amounts. 2) Spike-in material is added early in the process as to be present during technical sample manipulations throughout the entire ChIP procedure and library generation. 3) Spike-in material is present in small amounts so that library generation is not affected, and so that the final sequencing data is not dominated by tags originating from the spike-in material. 4) Spike-in material is uniquely identifiable in the final data set. 5) Spike-in material can be applied to ChIP reactions universally, independent of the nature of the experimental sample chromatin and independent of the experiment-specific antibody.

**Fig 3:**
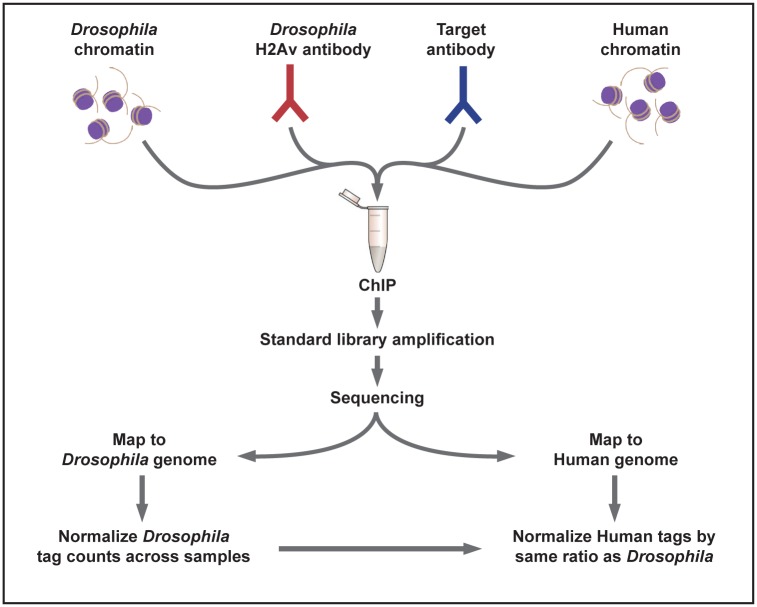
Schematic representation of the ChIP-seq spike-in protocol. ChIP-seq spike-in reactions are set up by adding the test chromatin of interest (human or other), the target antibody of interest, a small portion of *D*. *melanogaster* chromatin and the *D*. *melanogaster-*H2Av-specific antibody. The *D*. *melanogaster* spike-in chromatin is added in equal amounts and the H2Av antibody functions to pull down a small portion of the *D*. *melanogaster* chromatin in each reaction. After sequencing, tags are mapped to the genome corresponding to the test chromatin as well as to the *D*. *melanogaster* genome. The total number of tags uniquely mapping to the *D*. *melanogaster* genome are counted for each sample and used to generate correction factors (DMSO tags/inhibitor tags). The test chromatin tag counts are then normalized using the correction factors.

To satisfy these criteria we designed a *D*. *melanogaster* chromatin spike-in strategy that includes the addition of a *D*. *melanogaster* histone variant protein-specific antibody (Fig 3). The purpose of the *D*. *melanogaster*-specific antibody is to provide a means to reliably immunoprecipitate *D*. *melanogaster* chromatin from each reaction, which is then used as a reference for normalization. We chose an antibody against the *D*. *melanogaster*-specific histone variant *His2Av* (H2Av) as a control [29] since the antibody recognizing this histone variant does not cross-react with the histone H2A.Z variant protein from other species. First, a series of antibody titration ChIP experiments were carried out to ensure that H3K27me3 antibodies precipitate concentration-proportional amounts of DNA (S3 and S4 Figs). Next, ChIP-qPCR antibody titration experiments and ChIP-seq were carried out with the H2Av antibody and chromatin from the *D*. *melanogaster* S2 cell line. Results showed a significant enrichment pattern at specific genomic loci and across the *D*. *melanogaster* genome (S4 Fig). Moreover, the H2Av antibody did not precipitate human chromatin (S4 Fig), and thus did not show any genome-wide enrichment pattern in ChIP-seq (S4 Fig). These data confirm that the H2Av antibody uniquely precipitates the *D*. *melanogaster* reference chromatin.

To determine an appropriate starting amount of *D*. *melanogaster* chromatin to spike into the human ChIP reactions, a rational approach was used. The *D*. *melanogaster* genome is 27 times smaller than the human genome, thus a ratio of 27:1 of human to *D*. *melanogaster* chromatin would theoretically achieve equality in terms of genome copy number. Using this ratio, and assuming the antibodies immunoprecipitate the same amount of *D*. *melanogaster* and human chromatin per unit chromatin, a ChIP reaction that contains 30 μg of human chromatin should be spiked with 1.11 μg of *D*. *melanogaster* chromatin. Under these conditions a ChIP-seq experiment in which 50 million tags are sequenced should yield approximately 1.85 million *D*. *melanogaster* sequence tags. We scaled our *D*. *melanogaster* spike-in amount down to 750 ng with the goal of achieving approximately 1 million *D*. *melanogaster* sequence tags in the final data set.

To validate the spike-in strategy with ChIP-qPCR, *D*. *melanogaster* chromatin from S2 cells was mixed with chromatin from KARPAS-422 cells treated with DMSO or 1.5 μM CPI-360 for 8 days. Constant amounts of H3K27me3 and H2Av antibodies were used in each ChIP reaction and the enrichment levels of selected human and *D*. *melanogaster* genomic locations were determined by qPCR. As expected, the H3K27me3 enrichment at all inspected human genomic loci were significantly reduced by CPI-360 treatment (S5 Fig). However, ChIP using the H3K27me3-specific antibody, which recognizes and precipitates both human and *D*. *melanogaster* tri-methylated H3K27, did result in a marginal increase in enrichment at individual genomic loci from inhibitor treated cells. Presumably, the extreme reduction of H3K27me3 levels in the treated human samples leads to an increase in free H3K27me3 antibody, resulting in more *D*. *melanogaster* chromatin precipitated from the inhibitor treated cells.

### ChIP-seq Spike-In Normalization Reveals the Impact of EZH2 Inhibitors on Genome-Wide H3K27me3 Levels

**Fig 4:**
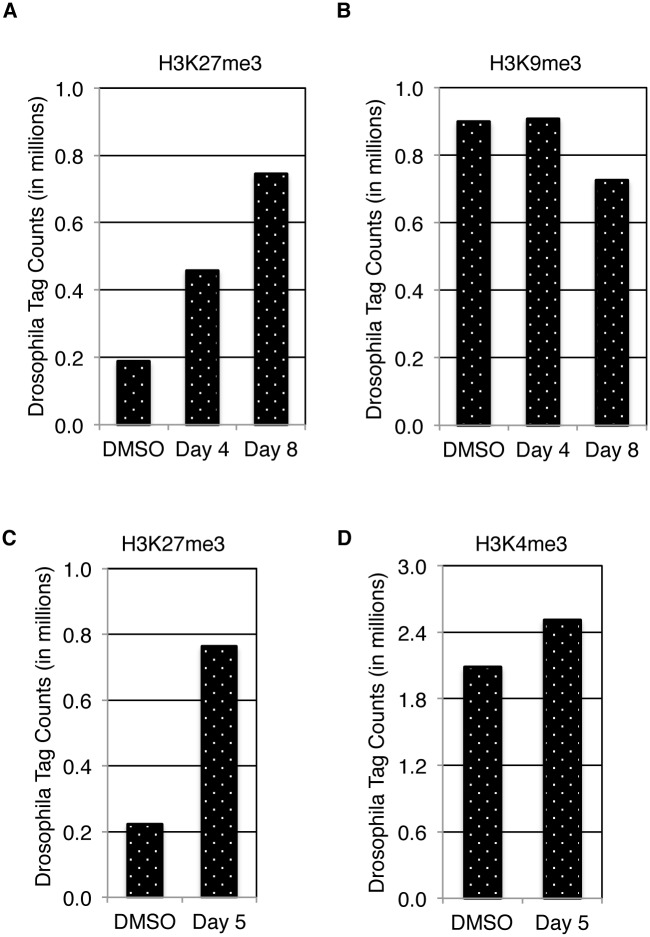
*D*. *melanogaster* tag counts from H3K27me3 ChIP-seq reactions are elevated in EZH2 inhibitor treated samples. H2Av bound regions of the *D*. *melanogaster* genome were determined using the H2Av antibody in ChIP-seq reactions containing *D*. *melanogaster* S2 or OSS chromatin. *D*. *melanogaster* tags from ChIP-seq spike-in reactions were mapped only to these pre-defined H2Av regions. **(A)** H3K27me3 ChIP-seq reactions with *D*. *melanogaster* spike-in in KARPAS-422 cells have a substantial increase in *D*. *melanogaster* tags in spike-in libraries prepared from CPI-360 treated cells both at 4 days and 8 days after treatment. **(B)** The increase was not observed in the control H3K9me3 reactions. **(C)** H3K27me3 ChIP-seq reactions with *D*. *melanogaster* spike-in in PC9 cells have a substantial increase in *D*. *melanogaster* tags in spike-in libraries prepared from GSK126 treated cells. **(D)** The substantial increase in tags was not observed in the control H3K4me3 ChIP-seq spike-in reactions.

ChIP-seq experiments were carried out using chromatin preparations and H3K27me3 antibody amounts as described in the Materials and Methods but with the addition of chromatin from *D*. *melanogaster* S2 or OSS cell lines and the H2Av antibody. While standard ChIP-seq protocols and analysis methods do not detect the global decreases in H3K27me3 throughout the genome following inhibition of EZH2 (Fig 1), our experimental design allows for an alternative data analysis: ChIP-seq reactions were processed using standard methods and final sequencing tags were mapped to both the human and the *D*. *melanogaster* genomes. Since the final *D*. *melanogaster* tag counts are the basis for the normalization we mapped the tags in two different ways. The first approach was designed based on our concern that one potential source of variation in our spike-in strategy could arise from cross-reactivity of the H3K27me3 antibody with *D*. *melanogaster* chromatin (S5 Fig). Specifically, when comparing samples such as DMSO and EZH2 inhibitor treatment that contain dramatically different levels of the target antigen, the effective antibody concentration available for enrichment of the *D*. *melanogaster* chromatin in one sample versus the other may skew the tag counts and affecting the normalization. To mitigate this possibility, we designed a *D*. *melanogaster* sequence tag mapping strategy that uses a reference genome that corresponds only to H2Av binding sites in *D*. *melanogaster* and is thus deficient for sites bound by the H3K27me3 antibody. Using this strategy only H2Av-enriched DNA is mapped, thus excluding *D*. *melanogaster* chromatin pulled down by the experimental antibody. Counting *D*. *melanogaster* tags in this manner resulted in increased tag numbers in EZH2 inhibitor-treated samples over DMSO-treated samples (Fig 4A and 4C and S6 Fig). For the second approach, we used the entire *D*. *melanogaster* genome as a reference genome, kept only tags that uniquely aligned and removed all duplicates. Using this approach, we also found that there were significantly more tags mapping to the *D*. *melanogaster* genome in H3K27me3 ChIP reactions containing chromatin from EZH2 inhibitor-treated cells (S7 Fig). Both of these mapping strategies showed similar increases in *D*. *melanogaster* tag counts in inhibitor treated samples thus both strategies are effective in revealing decreased ChIP-seq signal from inhibitor treated samples once correction factors are applied to the corresponding human tag counts (S3 Table).

**Fig 5:**
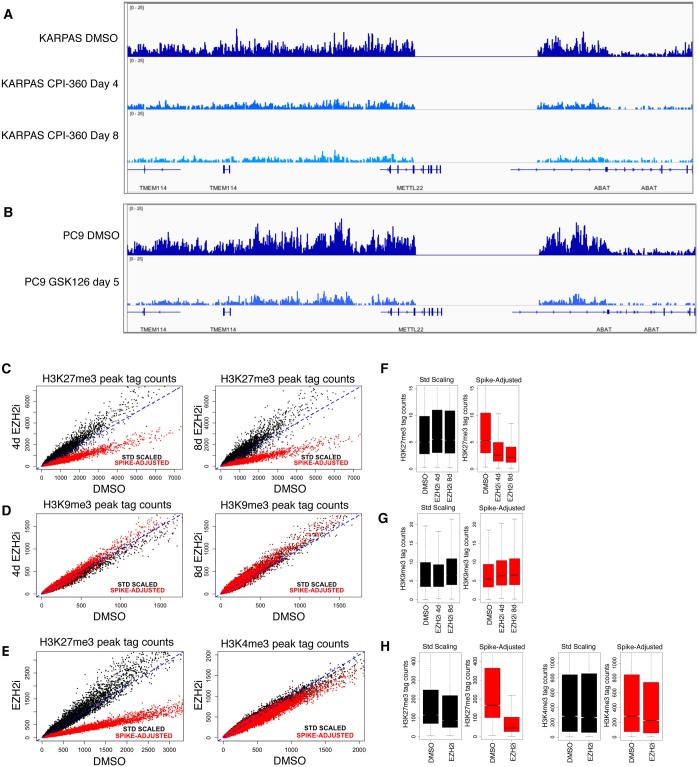
Spike-in normalization reveals the expected H3K27me3 decrease following EZH2 inhibition. **(A)** IGV browser image of spike-in normalized H3K27me3 ChIP-seq data from cells treated for 4 and 8 days with CPI-360. **(B)** Browser image of spike-in normalized H3K27me3 ChIP-seq data from cells treated for 5 days with GSK126. **(C)** Scatter plots representing the correlation of all H3K27me3 ChIP-seq peaks before and after CPI-360 treatment. Spike-in normalization dramatically decreases H3K27me3 signal in cells treated for 4 days and 8 days. (**D**) Scatter plots representing the correlation of all H3K9me3 ChIP-seq peaks before and after CPI-360 treatment. Spike-in normalization does not significantly affect H3K9me3 signal in cells treated with CPI-360 for 4 day and 8 days. (**E**) Scatter plots representing the correlation of all H3K27me3 and H3K4me3 ChIP-seq peaks before and after GSK126 treatment. Spike-in normalization dramatically decreases H3K27me3 signal in inhibitor treated cells but does not significantly affect H3K4me3 signal. **(F)** Box plots representing H3K27me3 ChIP-seq data sets from DMSO, 4 day and 8 day CPI-360 treated cells. (**G**) Box plots representing H3K9me3 ChIP-seq data sets from DMSO, 4 day and 8 day CPI-360 treated cells. **(H)** Box plots representing H3K27me3 and H3K4me3 ChIP-seq data sets from DMSO and GSK126 treated cells.

In subsequent experiments tag counts were derived from ‘H2Av-only’ *D*. *melanogaster* genome mapping and used to generate a correction factor (DMSO tags/inhibitor tags) (S4 Table). The correction factor was used to normalize the human tag counts, resulting in a significant reduction of mapped human tags in the inhibitor-treated H3K27me3 ChIP-seq data sets. CPI-360 treatment for 4 and 8 days led to a marked reduction in H3K27me3 levels across the genome in KARPAS-422 cells (Fig 5A), similar to what was observed in PC9 cells upon treatment with GSK126 (Fig 5B). Both compounds substantially reduced H3K27me3 levels at most H3K27me3 positive locations throughout the genome (Fig 5C and 5E). ChIP-seq data normalized using this strategy showed an approximate 4-fold reduction in H3K27me3 signal in CPI-360-treated cells and a 5-fold reduction in the H3K27me3 signal in GSK126-treated cells as compared to DMSO controls (Fig 5F and 5H), consistent with expectations based on western blot and mass spectrometry data. Importantly, these compound-induced H3K27me3 decreases were detected across multiple biological replicates (S8 Fig) but were not seen when standard analysis methods were employed.

In order to verify that the normalization to *D*. *melanogaster* sequence tags does not artificially introduce changes in ChIP-seq signals, we performed control experiments with antibodies against modifications that are not expected to change in response to EZH2 inhibitor treatment. H3K4me3 and H3K9me3 ChIP-seq were performed in PC9 cells treated with GSK126 and in KARPAS-422 cells treated with CPI-360 as compared to DMSO controls. The *D*. *melanogaster* tag counts in these control ChIP-seq reactions were more evenly distributed across samples than in the H3K27me3 ChIP reactions (Fig 4B and 4D). As a result, normalization to *D*. *melanogaster* sequence tag counts did not significantly change the data sets and no significant changes between DMSO and inhibitor treated samples were detected (Fig 5D, 5E, 5G and 5H). Therefore, normalization to *D*. *melanogaster* tag counts does not introduce artificial differences but rather reveals changes that reflect the true biology of the samples. Taken together the data show that the *D*. *melanogaster* chromatin/H2Av antibody spike-in approach is an effective strategy that reveals the specific reduction in H3K27me3 ChIP-seq signal in response to EZH2 inhibitor treatment.

## Discussion

ChIP-seq has emerged as a widely used and powerful technology for the mapping of transcription factor binding sites and histone modification occupancy across the genome. Soon after the development of the technique significant resources were invested to systematically catalogue transcription factor binding sites and histone modification patterns across a number of cell types. Led by the ENCODE consortium this effort was first focused on mapping multiple marks in a diverse set of cell lines [30]. However, these efforts did not include drug exposures that would significantly alter the profiles within a given cell type. As the field matures, questions regarding dynamic global changes in occupancy upon manipulation of a specific cellular context have become more important. The focus on these more complex experimental models has led to the recognition of limitations in current ChIP-seq protocols and analysis methods [31]. For example, standard ChIP-seq analysis does not reveal comparative differences when looking at global changes in highly prevalent histone modifications, such as decreased H3K27me3 occupancy following treatment with EZH2 inhibitors.

The only reliable way to overcome bias that can be introduced at various stages of the ChIP-seq procedure is to add a known standard (i.e. spike-in) into all samples and determine if the standard is differentially affected during processing. Recently, ChIP-seq spike-in strategies have been described [8, 25] that are similar to the approach we describe herein. The published methods propose a small amount of human chromatin to be spiked into mouse chromatin IP reactions, or a small amount of *D*. *melanogaster* chromatin to be spiked into human chromatin IP reactions. These methods require the experimental ChIP antibody to immunoprecipitate both the test chromatin and the spiked-in reference chromatin and are therefore dependent on species cross-reactivity of the antibody. The approach we have developed uses *D*. *melanogaster* chromatin, which is not highly similar to human or mouse, resulting in definitive mapping of tags to the correct species. The addition of a second, *D*. *melanogaster*-specific antibody is a key differentiating feature of our approach, which increases robustness and versatility. We determined that the H2Av antibody is adequate for ChIP-seq normalization when added with the *D*. *melanogaster* reference chromatin. In this approach the experimental antibody does not need to cross-react with the reference chromatin providing a widely applicable normalization approach which has the potential to be applied to non-histone targets.

From a technical perspective the *D*. *melanogaster* chromatin spike-in is simple. ChIP protocols remain unchanged except for addition of the *D*. *melanogaster*-specific H2Av antibody and a small amount of *D*. *melanogaster* chromatin from a common stock. Library generation and DNA sequencing are performed as usual as is the mapping of human sequence tags. Normalization of human tags is based on differences in *D*. *melanogaster* tag counts using an approach to map tags uniquely to the pre-defined H2Av binding sites or the alternate approach of using all tags that uniquely map across the entire *D*. *melanogaster* genome. In the case of the H3K27me3 antibody, it recognizes both human and *D*. *melanogaster* chromatin. Thus, it is possible to re-analyze the data according to a previously published method [8] where normalization is based on *D*. *melanogaster* tag counts using only H3K27me3-specific regions of the *D*. *melanogaster* genome. Analysis of our data using this method resulted in a correction factor between DMSO and EZH2 inhibitor-treated samples that was similar to those obtained with our H2Av method (S3 and S4 Tables). These results validate our methodology and suggest that both methods are adequate for H3K27me3 normalization. However, the cross-reactivity of the H3K27me3 antibody may affect the total amount of precipitated *D*. *melanogaster* chromatin, and thus constitutes a potential liability (S5 Fig) which was addressed herein by normalizing to *D*. *melanogaster* ‘H2Av-only’ tag counts. Methods such as Orlando et al. critically depend on cross-reactivity of the experimental antibody and are potentially more prone to inadequate normalization since there is less opportunity to keep the amount of precipitated *D*. *melanogaster* chromatin constant. One may alleviate this concern by ensuring that the amount of the experimental antibody is always in excess, and thus always precipitates the maximum amount of reference chromatin. However, target abundance dictates the required amount of antibody needed and re-establishing appropriate antibody concentrations for each target is a time-consuming process and may be technically difficult. Additionally, antibody excess potentially increases background, which may influence data analysis and normalization.

While Orlando et al. mix human and *D*. *melanogaster* cells and process them jointly, our method uses individual chromatin preparations that are mixed using a defined amount of human and *D*. *melanogaster* chromatin at a ratio of approximately 40:1. We believe that this approach has several potential advantages: (1) One can prepare a large amount of *D*. *melanogaster* chromatin and avoid batch to batch variations that may arise from repeatedly growing *D*. *melanogaster* cells along with culturing human cells for a given experiment. (2) *D*. *melanogaster* and human cells behave differently during chromatin processing. For instance, different sonication conditions are used to adequately fragment chromatin from both species. Even for human cells, sonication conditions may vary depending on cell type. If human and *D*. *melanogaster* cells are mixed, the human cell-specific conditions may be inadequate for *D*. *melanogaster* cells. Processing the cells separately will ensure that the quality of the reference standard is independent of human cell processing conditions.

Ultimately, one of the major problems contributing to the inability of ChIP-seq to detect global changes in binding or histone PTM occupancy seemingly occurs at library generation. Our DNA quantitation data show that for inhibitor treated cells there is less DNA going into library generation protocols (S1 Table). However, library yields are similar and loci-specific qPCR amplification from the library shows that inhibitor induced reductions are no longer detectable. A seemingly obvious solution to this problem is to reduce PCR cycle numbers during the amplification of the library. Unfortunately, even if quantitative differences in libraries, which reflect the differences in pre-library ChIP DNA, are maintained they will be lost during cluster generation in preparation for sequencing. In short, when multiplexing multiple barcoded libraries in a single sequencing run, the goal of the researcher is to sequence approximately the same number of tags per library. Therefore, careful quantitation of the library is undertaken as to assure equal cluster density for each sample. This universally accepted practice results in the loss of global changes when comparing two ChIP-seq data sets, even if library yields accurately capture the differences. A similar phenomenon has also been discussed by Loven et al. in RNA-Seq experiments and reinforces the need to use spike-in controls in genome-wide applications such as ChIP-seq and RNA-seq.

When designing the spike-in approach we rationalized that the ideal number of *D*. *melanogaster* tags would be 1 million tags from a 50 million-tag experiment, assuming that the antibodies pull down a similar fraction of chromatin in each species. This number would be enough for reliable normalization but would not negatively impact the overall tag count from the experimental samples. The initial experiments contained 750 ng of *D*. *melanogaster* chromatin resulting in approximately 1.2 million *D*. *melanogaster* tags in untreated PC9 cells. However, in the first experiment in KARPAS-422 DMSO-treated cells, tag counts were only 102,000, well below the target of 1 million. In the second experiment we increased the amount of *D*. *melanogaster* spike-in chromatin to 3.7 μg in KARPAS-422 cells, resulting in *D*. *melanogaster* tag counts of 831,000. One of the reasons why more spike-in chromatin is required in KARPAS-422 cells than in PC9 cells to achieve adequate tag counts, is that KARPAS-422 cells harbor a mono-allelic mutation in EZH2 which leads to increased global H3K27me3 levels as compared to PC9. As a consequence, H3K27me3-enriched DNA from KARPAS-422 cells is more prominent in the final ChIP-DNA population, thus the ratio of ChIP DNA originating from the spike-in compared to ChIP DNA originating from H3K27me3 enriched human chromatin is lower than that from PC9 cells. Importantly, we observed that using antibodies against less abundant histone modifications or transcription factors in ChIP-seq reactions containing 750 ng of *D*. *melanogaster* chromatin may result in *D*. *melanogaster* tag counts exceeding 30% of the total tags in the final sequencing data set. Therefore, different contexts may require different amounts of *D*. *melanogaster* chromatin in order to achieve a reasonable number of *D*. *melanogaster* tags in the final data set. In summary, the amount of spike-in chromatin required to achieve 1 million *D*. *melanogaster* sequence tags varies depending on the cell type, the prevalence of the target and the quality of the experimental antibody.

In conclusion, the ChIP-seq spike-in method described here was designed to be a broadly applicable solution for ChIP-seq normalization. This method overcomes a challenging and persistent barrier to our understanding of the mechanisms by which drugs targeting epigenetic modifying proteins may exert their clinical effect. EZH2 inhibitors have been available to the research community for several years, yet comparative H3K27me3 ChIP-seq profiles from DMSO and EZH2 inhibitor-treated disease-relevant cell models have not been broadly explored. Our spike-in system and normalization strategy used with EZH2 inhibitor treated samples revealed global reductions in H3K27me3 ChIP-seq signal in inhibitor treated samples. We expect that the adoption of this ChIP-seq spike-in normalization approach or other similar strategies [25, 8] will reveal many more previously undetected differences in chromatin states and chromatin factor occupancy, thus adding to the power of ChIP-seq as a biology discovery tool.

## Supporting Information

S1 Fig EZH2 inhibition reduces global levels of H3K27me3 but not of other histone modifications. (A) Reduced global H3K27me3 levels were observed in PC9 cells treated with 1 μM of GSK126 for 5 days. Immunoprecipitation using anti-H3K27me3 followed by immunoblotting with anti-H3 shows reduced H3K27me3 levels in inhibitor-treated cells. (B) Mass spectrometry analysis of histone H3K27 and H3K9 methylation abundance in CPI-360-treated (0.625, 2.5 and 10 μM) KARPAS-422 cells compared to DMSO treated controls. (C) Mass spectrometry analysis of histone H3K27 and H3K4 methylation abundance in GSK126-treated (1 μM) PC9 cells compared to DMSO-treated controls. (D) The KARPAS DMSO samples from duplicate H3K27me3 ChIP-seq experiments were compared do demonstrate the consistency of the ChIP-seq protocol. The biological replicates display a correlation coefficient of 0.985. (E) The PC9 DMSO samples from duplicate H3K27me3 ChIP-seq experiments were compared do demonstrate the consistency of our ChIP-seq protocol. These biological replicates display a correlation coefficient of 0.998.
(PDF)

S2 Fig Control histone modifications, H3K4me3 and H3K9me3, do not change following EZH2 inhibition. **(A)** ChIP-qPCR was performed using chromatin from KARPAS-422 cells treated with the EZH2 inhibitor CPI-360. H3K9me3 occupancy did not change at the ZNF274 and ZNF829 positive control genes after treatment. **(B)** ChIP-qPCR was performed using chromatin from PC9 cells treated with the EZH2 inhibitor GSK126. H3K4me3 occupancy did not change at the ACTB and GAPDH promoters after treatment.
(PDF)

S3 Fig Titration of H3K27me3 antibody using different amounts of human chromatin. Different chromatin amounts (12 and 6 μg) were used in ChIP reactions with different anti-H3K27me3 antibody amounts (250–8000 ng). ChIP DNA was analyzed by qPCR at indicated promoters. Data is represented as mean enrichment from two independent experiments and qPCRs carried out in triplicate ±SD. A region in the *U6-5* gene was used as negative control for H3K27me3 occupancy. qPCR results showed that H3K27me3 occupancy is proportional to the amount of H3K27me3 antibody used in ChIP reactions within the tested range (250 ng to 8 μg antibody/ChIP).
(PDF)

S4 Fig The H2Av antibody does not cross react with human chromatin in ChIP. Titration of H2Av and H3K27me3 antibodies with *D*. *melanogaster* **(A)** and human **(B)** chromatin. Antibody amounts used in ChIP reactions are indicated below the graphs. Chromatin amounts of 2.85 μg of *D*. *melanogaster* and 12 μg of human chromatin were used in each ChIP-reaction. ChIP DNA was analyzed by qPCR at indicated gene promoters. Data is represented as mean enrichment from two independent experiments and qPCRs carried out in triplicate ±SD. *DUntr3L* was used as negative control for H2Av occupancy. Results showed an antibody concentration-dependent increase of both H2Av and H3K27me3 occupancy at indicated *D*. *melanogaster* promoters. In ChIP reactions containing human chromatin, an antibody concentration-dependent increase in H3K27me3 at indicated human promoters was detected while H2Av ChIPs did not result in enrichment over background. **(C)** H2Av ChIP-seq data from reactions containing chromatin from only the *D*. *melanogaster* S2 cell line. The 13 million base pair region depicted shows hundreds of peaks detected on chromosome 2R. **(D)** H2Av ChIP-seq data from a reaction containing chromatin from only the human cell line PC9. Depicted are 95 million base pairs from human chromosome 5. The H2Av antibody does not cross react with human chromatin therefore no peaks are detected.
(PDF)

S5 Fig ChIP-qPCR validation of the spike-in strategy. Chromatin from DMSO or CPI-360 treated KARPAS-422 cells were mixed with *D*. *melanogaster* S2 chromatin for H3K27me3/H2Av ChIP. qPCR at different human and *D*. *melanogaster* genomic loci was performed to evaluate enrichment. U6-5 promoter was selected as the H3K27me3 negative locus in KARPAS-422 cells. CPI-360 treatment resulted in a significant decrease in H3K27me3 signal at human genes and slightly increased enrichment of *D*. *melanogaster* genes. Data represent the mean of three ChIP experiments with qPCR carried out in triplicates ±SEM. *Wilcoxon signed rank test.
(PDF)

S6 Fig *D*. *melanogaster* tag counts from H3K27me3 ChIP-seq, replicate 2. H2Av bound regions of the *D*. *melanogaster* genome were determined using the H2Av antibody in ChIP-seq reactions containing *D*. *melanogaster* S2 or OSS chromatin. *D*. *melanogaster* tags from ChIP-seq spike-in reactions were mapped only to these pre-defined H2Av regions. The trends in this replicate are similar to those shown in Fig 4. **(A)** H3K27me3 ChIP-seq reactions using *D*. *melanogaster* spike-in in KARPAS-422 cells results in an increase in *D*. *melanogaster* tags mapping in CPI-360 treated cells both at 4 days and 8 days after treatment. **(B)** H3K27me3 ChIP-seq reactions using *D*. *melanogaster* spike-in in PC9 cells results in an increase in *D*. *melanogaster* tags mapping in GSK126 treated cells.
(PDF)

S7 Fig *D*. *melanogaster* tag counts based on mapping across the entire *D*. *melanogaster* genome. Elevated *D*. *melanogaster* tag counts are observed in EZH2 inhibitor treated samples in H3K27me3 ChIP-seq spike-in reactions when using tags mapped across the entire *D*. *melanogaster* genome. Data is similar to the strategy of mapping to only the pre-defined H2Av regions that is presented in fig 4. **(A)** H3K27me3 ChIP-seq spike-in reactions using S2 *D*. *melanogaster* chromatin in KARPAS-422 cells. **(B)** H3K9me3 ChIP-seq spike-in reactions using S2 *D*. *melanogaster* chromatin in KARPAS-422 cells. **(C)** H3K27me3 ChIP-seq spike-in reactions using OSS *D*. *melanogaster* chromatin in PC9 cells. **(D)** H3K4me3 ChIP-seq spike-in reactions using OSS *D*. *melanogaster* chromatin in PC9.
(PDF)

S8 Fig Biological replicates show similar effects of spike-in normalization on ChIP-seq data. **(A)** Chromatin from DMSO or CPI-360-treated KARPAS-422 cells and **(B)** chromatin from DMSO or GSK126-treated PC9 cells were mixed with *D*. *melanogaster* S2 and OSS chromatin respectively for H3K27me3/H2Av and H3K4me3/H2Av ChIPs. ChIP-seq was carried out on a biological replicate distinct from the experiment shown in Fig 5C and 5E. A significant decrease in H3K27me3 signal was detected using spike-in adjusted (SPIKE ADJUSTED; red) but not with standard (STD SCALED; black) normalization methods. The relative reduction of H3K27me3 signal varies across biological replicates (compare Fig 5C with S8A Fig and Fig 5E with S8B Fig) which likely reflect differences in how frequently the cells replicate within a given treatment period.
(PDF)

S9 Fig Uncropped images of blots for CPI-360-treated cells. (PDF)

S10 Fig Uncropped images of blots for GSK126-treated cells. (PDF)

S1 Methods (DOCX)

S1 Table Total DNA mass precipitated from H3K27me3 ChIP reactions. ChIP DNA was quantified using Qubit fluorometric quantitation. Total immunoprecipitated DNA mass and fold change after EZH2 inhibitor treatment are presented.
(PDF)

S2 Table Summary of ChIP-seq library yields. ChIP-seq libraries were PCR amplified for 15 cycles. ChIP-seq library yields were measured using a NanoDrop spectrophotometer. Yields within groups of treated and untreated cells were similar.
(PDF)

S3 Table Correction factors for EZH2-inhibitor treated versus DMSO treated ChIP-seq samples. Normalization factors were calculated based on tags mapping to the entire dm3 genome (168,736,537 bp), H2Av peak regions (15,942,729 bp) or H3K27me3 peak regions (34,740,761 bp). Normalization factors were calculated from two independent ChIP-seq spike-in campaigns (replicate 1, 2). For KARPAS-422 cells, H3K27me3 ChIP-seq was carried out on cells that were treated with DMSO or 1.5 μM CPI-360 for 8 days and H3K9me3 ChIP-seq from cells treated for 4 and 8 days. For PC9 cells, H3K27me3 and H3K4me3 ChIP-seq was carried out on cells that were treated with DMSO or 1 μM GSK126 for 5 days. Numbers represent the ratio of human (top) and *D*. *melanogaster* (bottom) reads in treated versus control samples.
(PDF)

S4 Table ChIP sequencing tag numbers and correction factor calculations. (XLSX)
