# Supplementary figures and images for: An Alternative Approach to ChIP-Seq Normalization Enables Detection of Genome-Wide Changes in Histone H3 Lysine 27 Trimethylation upon EZH2 Inhibition
Source: PLoS One. 2016 Nov 22;11(11):e0166438. doi: 10.1371/journal.pone.0166438 (PMC5119738; doi:10.1371/journal.pone.0166438)

**A**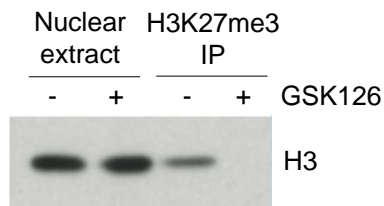**B**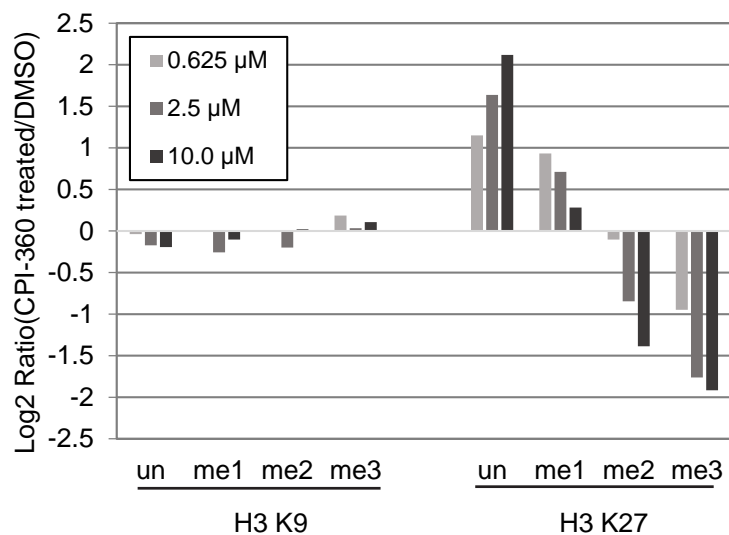**C**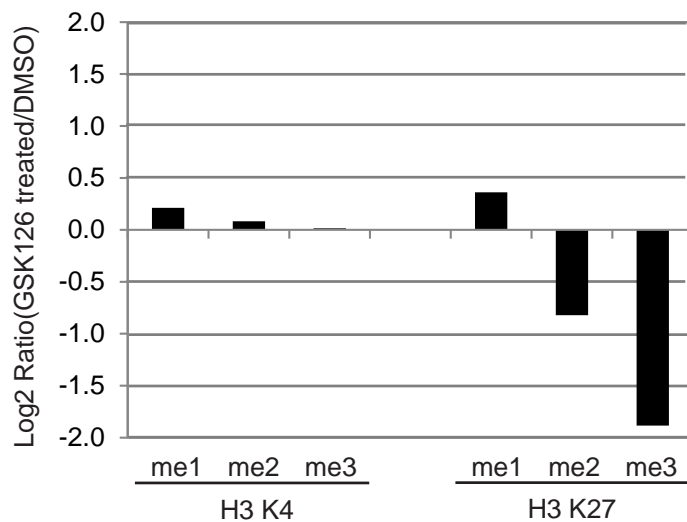**D**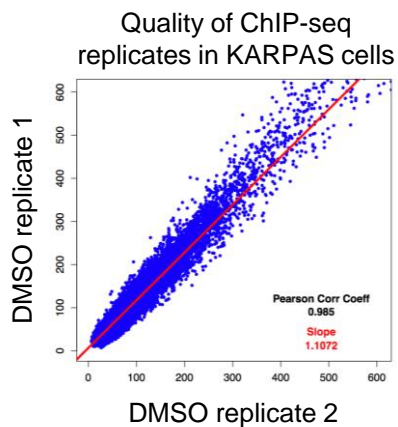**E**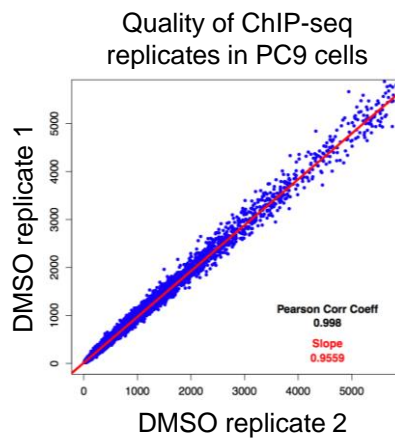

Supplement: S1 Fig — (A) Reduced global H3K27me3 levels were observed in PC9 cells treated with 1 μM of GSK126 for 5 days. Immunoprecipitation using anti-H3K27me3 followed by immunoblotting with anti-H3 shows reduced H3K27me3 levels in inhibitor-treated cells. (B) Mass spectrometry analysis of histone H3K27 and H3K9 methylation abundance in CPI-360-treated (0.625, 2.5 and 10 μM) KARPAS-422 cells compared to DMSO treated controls. (C) Mass spectrometry analysis of histone H3K27 and H3K4 methylation abundance in GSK126-treated (1 μM) PC9 cells compared to DMSO-treated controls. (D) The KARPAS DMSO samples from duplicate H3K27me3 ChIP-seq experiments were compared do demonstrate the consistency of the ChIP-seq protocol. The biological replicates display a correlation coefficient of 0.985. (E) The PC9 DMSO samples from duplicate H3K27me3 ChIP-seq experiments were compared do demonstrate the consistency of our ChIP-seq protocol. These biological replicates display a correlation coefficient of 0.998. (PDF) [file pone.0166438.s001.pdf]

**A**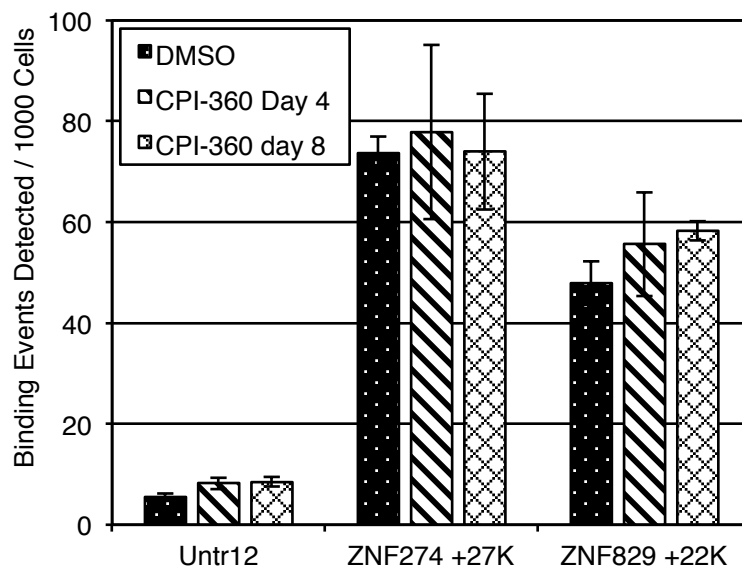**B**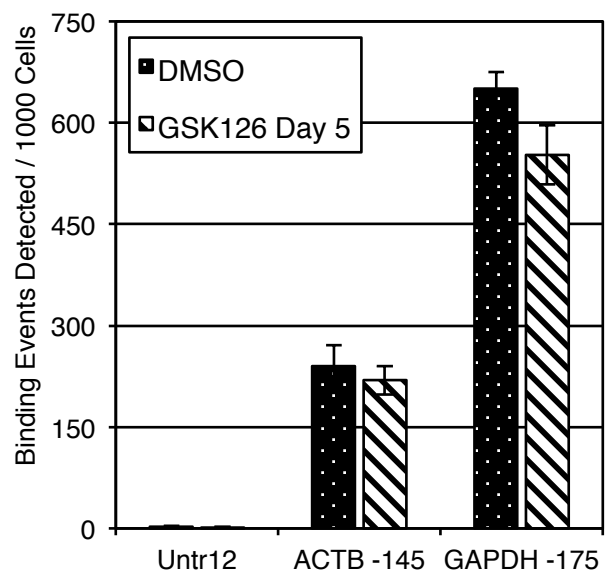

Supplement: S2 Fig — (A) ChIP-qPCR was performed using chromatin from KARPAS-422 cells treated with the EZH2 inhibitor CPI-360. H3K9me3 occupancy did not change at the ZNF274 and ZNF829 positive control genes after treatment. (B) ChIP-qPCR was performed using chromatin from PC9 cells treated with the EZH2 inhibitor GSK126. H3K4me3 occupancy did not change at the ACTB and GAPDH promoters after treatment. (PDF) [file pone.0166438.s002.pdf]

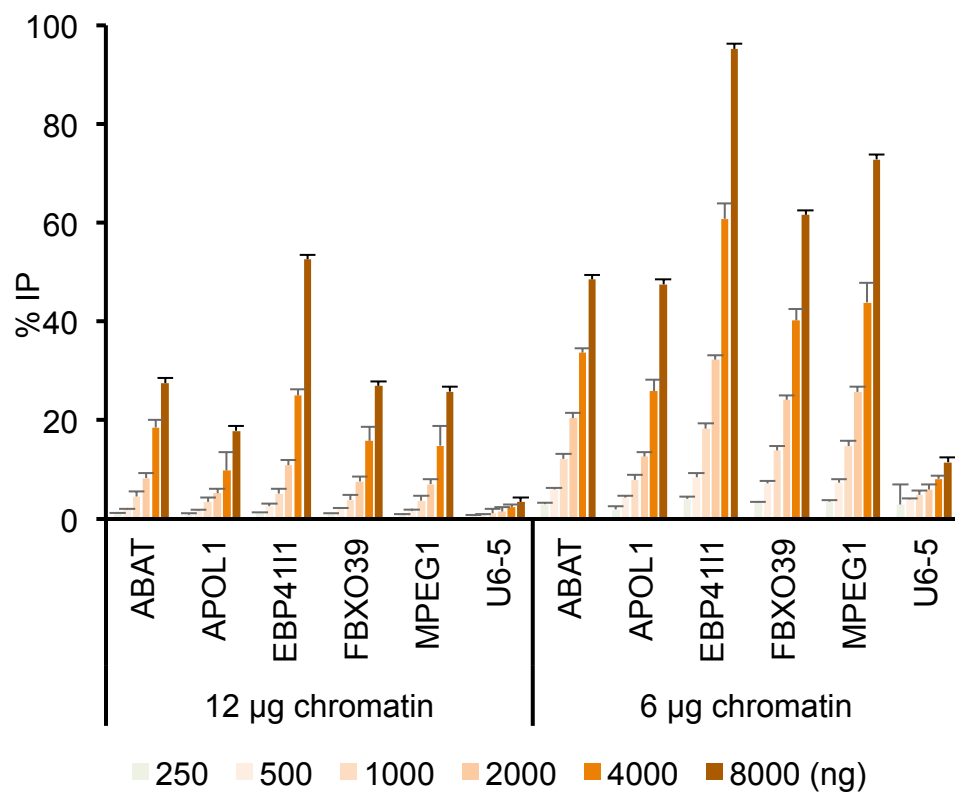

Supplement: S3 Fig — Different chromatin amounts (12 and 6 μg) were used in ChIP reactions with different anti-H3K27me3 antibody amounts (250–8000 ng). ChIP DNA was analyzed by qPCR at indicated promoters. Data is represented as mean enrichment from two independent experiments and qPCRs carried out in triplicate ±SD. A region in the U6-5 gene was used as negative control for H3K27me3 occupancy. qPCR results showed that H3K27me3 occupancy is proportional to the amount of H3K27me3 antibody used in ChIP reactions within the tested range (250 ng to 8 μg antibody/ChIP). (PDF) [file pone.0166438.s003.pdf]

**A**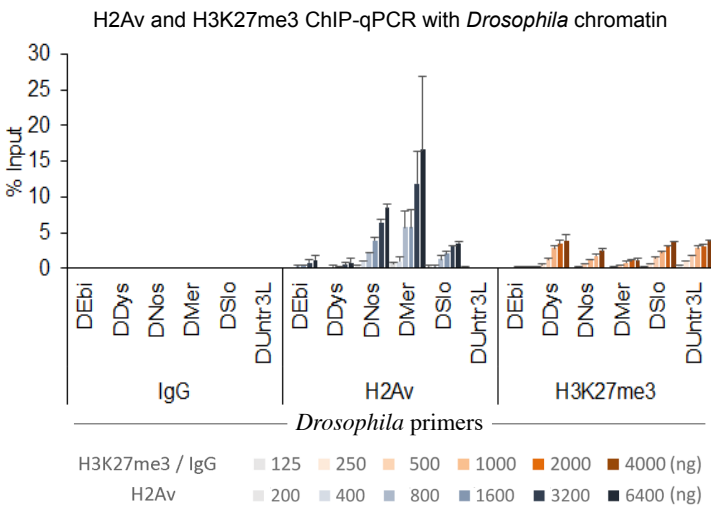**B**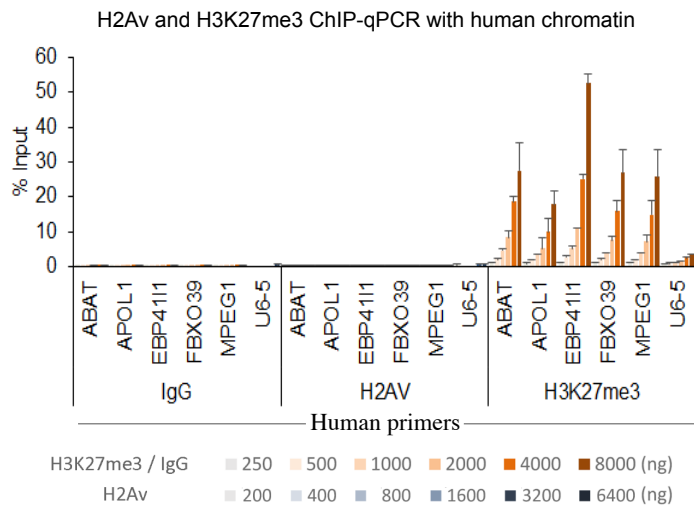

H2Av ChIP-Seq with *Drosophila* chromatin

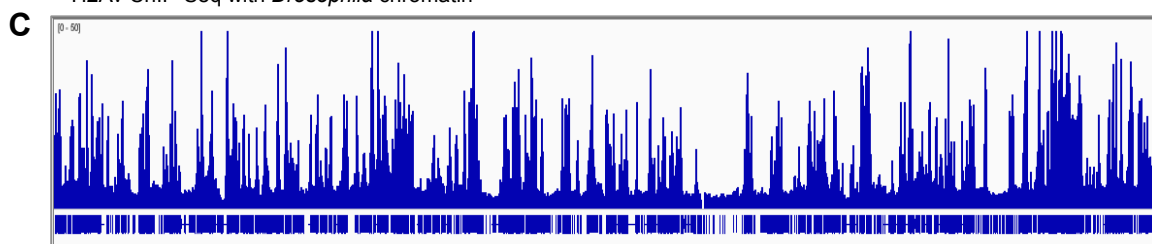

H2Av ChIP-Seq with human chromatin

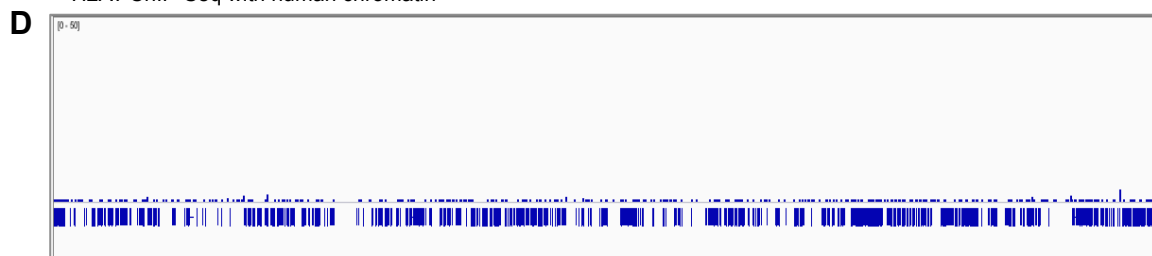

Supplement: S4 Fig — Titration of H2Av and H3K27me3 antibodies with D. melanogaster (A) and human (B) chromatin. Antibody amounts used in ChIP reactions are indicated below the graphs. Chromatin amounts of 2.85 μg of D. melanogaster and 12 μg of human chromatin were used in each ChIP-reaction. ChIP DNA was analyzed by qPCR at indicated gene promoters. Data is represented as mean enrichment from two independent experiments and qPCRs carried out in triplicate ±SD. DUntr3L was used as negative control for H2Av occupancy. Results showed an antibody concentration-dependent increase of both H2Av and H3K27me3 occupancy at indicated D. melanogaster promoters. In ChIP reactions containing human chromatin, an antibody concentration-dependent increase in H3K27me3 at indicated human promoters was detected while H2Av ChIPs did not result in enrichment over background. (C) H2Av ChIP-seq data from reactions containing chromatin from only the D. melanogaster S2 cell line. The 13 million base pair region depicted shows hundreds of peaks detected on chromosome 2R. (D) H2Av ChIP-seq data from a reaction containing chromatin from only the human cell line PC9. Depicted are 95 million base pairs from human chromosome 5. The H2Av antibody does not cross react with human chromatin therefore no peaks are detected. (PDF) [file pone.0166438.s004.pdf]

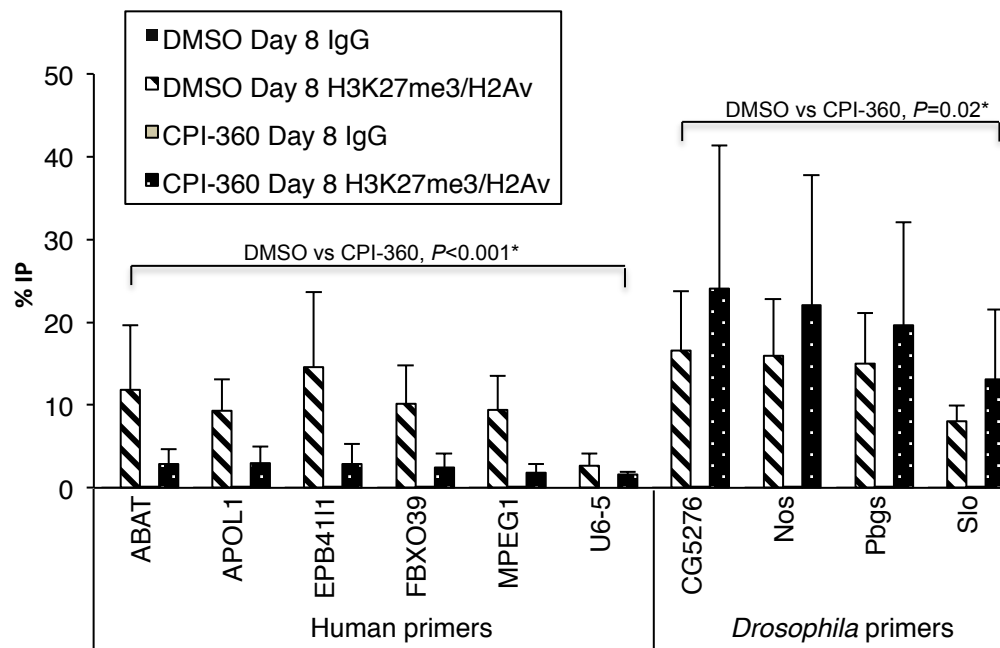

Supplement: S5 Fig — Chromatin from DMSO or CPI-360 treated KARPAS-422 cells were mixed with D. melanogaster S2 chromatin for H3K27me3/H2Av ChIP. qPCR at different human and D. melanogaster genomic loci was performed to evaluate enrichment. U6-5 promoter was selected as the H3K27me3 negative locus in KARPAS-422 cells. CPI-360 treatment resulted in a significant decrease in H3K27me3 signal at human genes and slightly increased enrichment of D. melanogaster genes. Data represent the mean of three ChIP experiments with qPCR carried out in triplicates ±SEM. *Wilcoxon signed rank test. (PDF) [file pone.0166438.s005.pdf]

**A**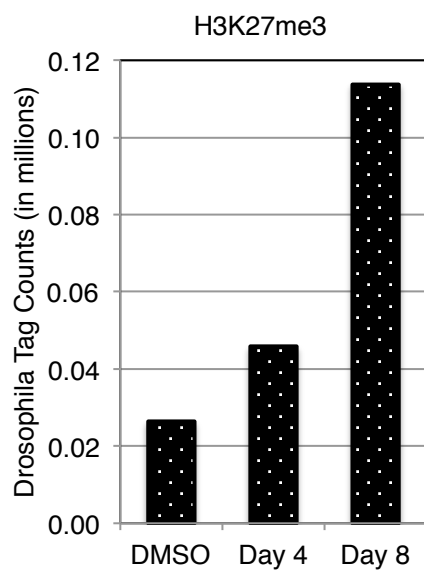**B**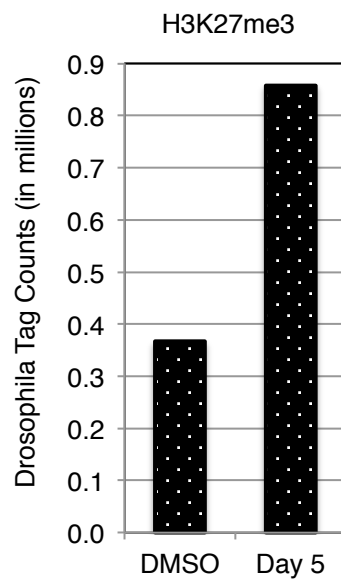

Supplement: S6 Fig — H2Av bound regions of the D. melanogaster genome were determined using the H2Av antibody in ChIP-seq reactions containing D. melanogaster S2 or OSS chromatin. D. melanogaster tags from ChIP-seq spike-in reactions were mapped only to these pre-defined H2Av regions. The trends in this replicate are similar to those shown in Fig 4. (A) H3K27me3 ChIP-seq reactions using D. melanogaster spike-in in KARPAS-422 cells results in an increase in D. melanogaster tags mapping in CPI-360 treated cells both at 4 days and 8 days after treatment. (B) H3K27me3 ChIP-seq reactions using D. melanogaster spike-in in PC9 cells results in an increase in D. melanogaster tags mapping in GSK126 treated cells. (PDF) [file pone.0166438.s006.pdf]

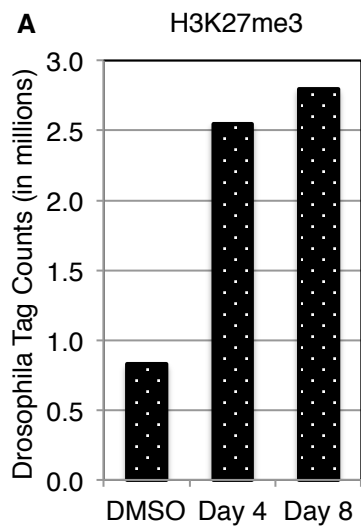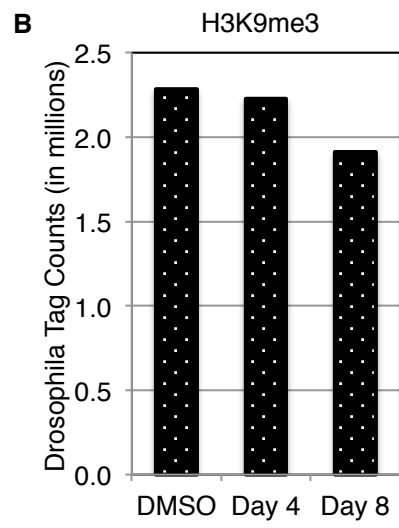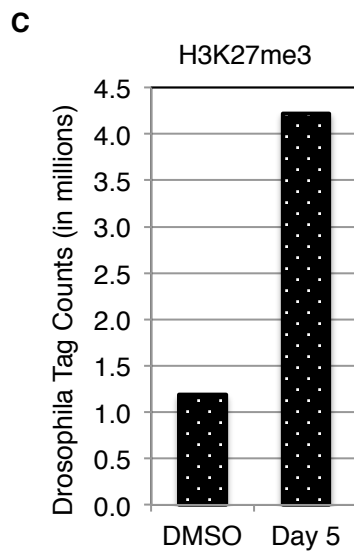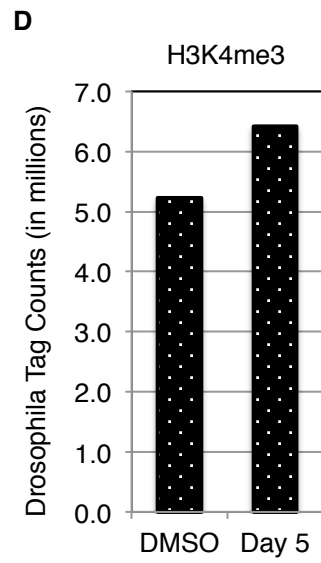

Supplement: S7 Fig — Elevated D. melanogaster tag counts are observed in EZH2 inhibitor treated samples in H3K27me3 ChIP-seq spike-in reactions when using tags mapped across the entire D. melanogaster genome. Data is similar to the strategy of mapping to only the pre-defined H2Av regions that is presented in fig 4. (A) H3K27me3 ChIP-seq spike-in reactions using S2 D. melanogaster chromatin in KARPAS-422 cells. (B) H3K9me3 ChIP-seq spike-in reactions using S2 D. melanogaster chromatin in KARPAS-422 cells. (C) H3K27me3 ChIP-seq spike-in reactions using OSS D. melanogaster chromatin in PC9 cells. (D) H3K4me3 ChIP-seq spike-in reactions using OSS D. melanogaster chromatin in PC9. (PDF) [file pone.0166438.s007.pdf]

**A**

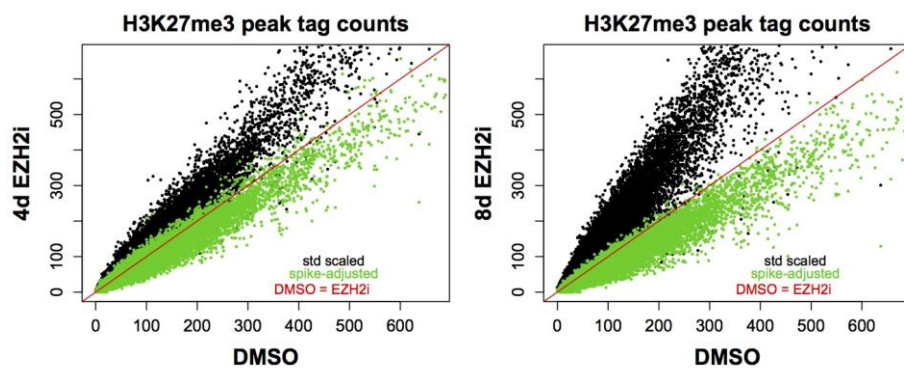

**B**

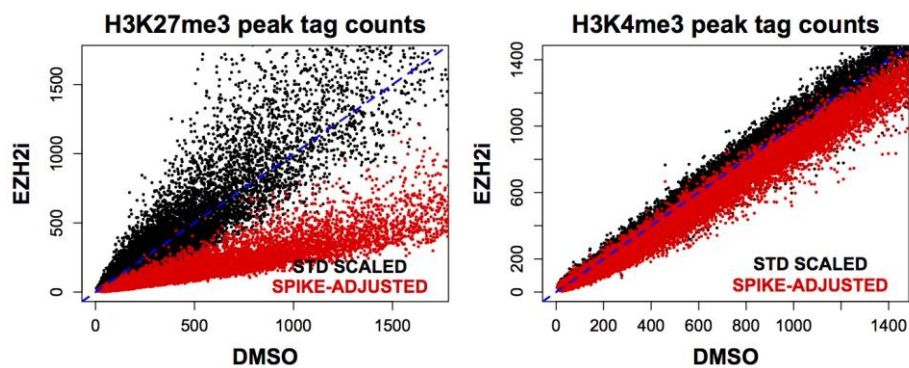

Supplement: S8 Fig — (A) Chromatin from DMSO or CPI-360-treated KARPAS-422 cells and (B) chromatin from DMSO or GSK126-treated PC9 cells were mixed with D. melanogaster S2 and OSS chromatin respectively for H3K27me3/H2Av and H3K4me3/H2Av ChIPs. ChIP-seq was carried out on a biological replicate distinct from the experiment shown in Fig 5C and 5E. A significant decrease in H3K27me3 signal was detected using spike-in adjusted (SPIKE ADJUSTED; red) but not with standard (STD SCALED; black) normalization methods. The relative reduction of H3K27me3 signal varies across biological replicates (compare Fig 5C with S8A Fig and Fig 5E with S8B Fig) which likely reflect differences in how frequently the cells replicate within a given treatment period. (PDF) [file pone.0166438.s008.pdf]

| DMSO | CPI-360 |    |
|------|---------|----|
|      | D4      | D8 |

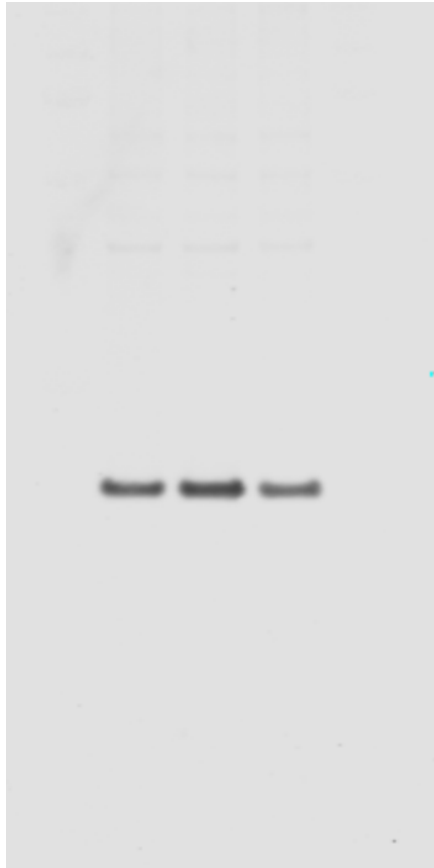

Histone H3 blot

| DMSO | CPI-360 |    |
|------|---------|----|
|      | D4      | D8 |

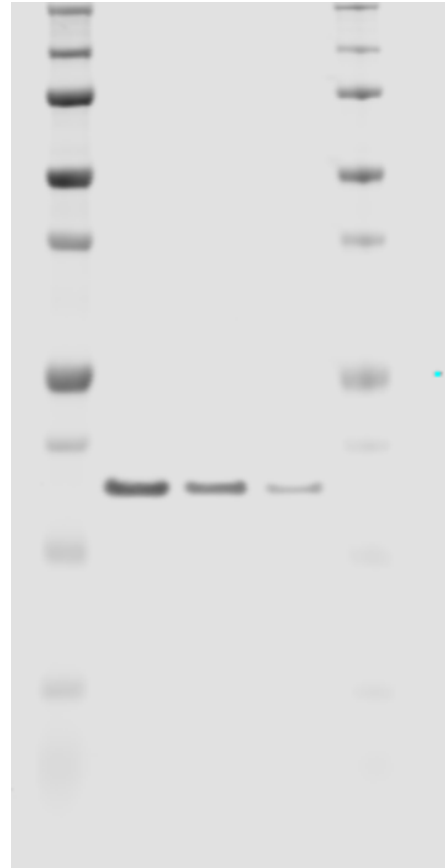

H3K27me3 blot

Supplement: S9 Fig — (PDF) [file pone.0166438.s009.pdf]

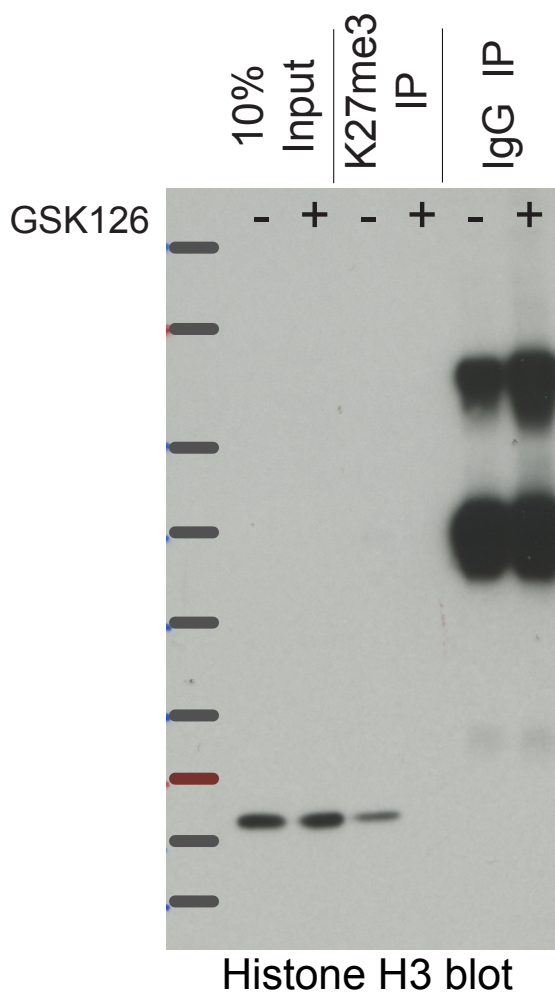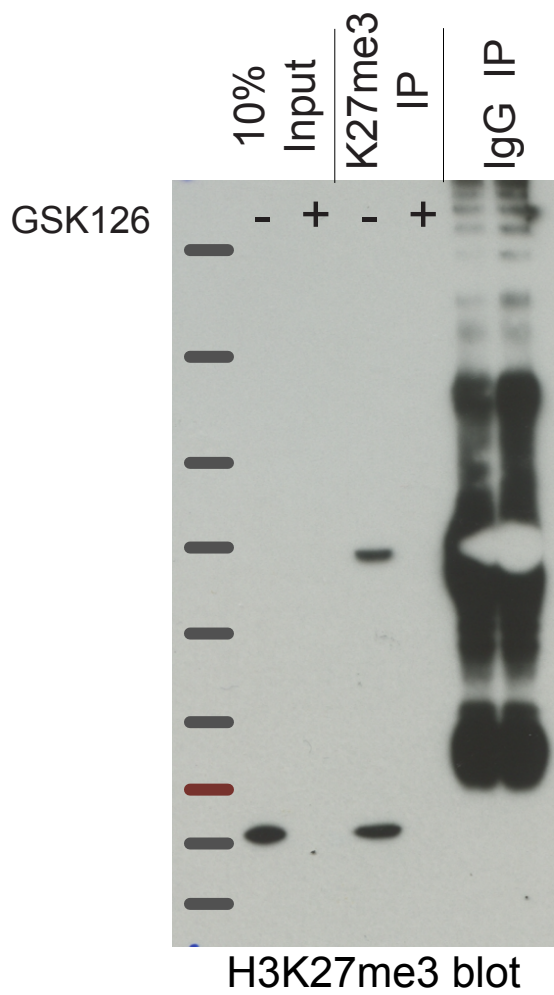

Supplement: S10 Fig — (PDF) [file pone.0166438.s010.pdf]
