# Supplementary material for: An Alternative Approach to ChIP-Seq Normalization Enables Detection of Genome-Wide Changes in Histone H3 Lysine 27 Trimethylation upon EZH2 Inhibition
Source: PLoS One. 2016 Nov 22;11(11):e0166438. doi: 10.1371/journal.pone.0166438 (PMC5119738; doi:10.1371/journal.pone.0166438)
Supplement: S1 Methods — (DOCX) [file pone.0166438.s011.docx]

**Supporting Information**

**Supporting Methods**

**Immunoprecipitation (IP) and Western Blot**

Immunoprecipitations were performed with nuclear extracts from DMSO or GSK126 treated PC9 cells using the Nuclear Complex Co-IP kit (Active Motif #54001) as described in manufacturer’s instructions. Briefly, cells were resuspended in 1x hypotonic buffer, incubated on ice for 15 minutes, then centrifuged at 14000x rpm for 30 seconds following addition of detergent. Nuclear pellets were resuspended in buffer (0.5 M NaCl, 0.1% SDS, 1% Triton) and sonicated for 30 cycles of 20 seconds ON and 30 seconds OFF. KARPAS-422 cell extracts were prepared from each of the cell lines by first separating the cytoplasmic fraction using buffer A (10 mM Tris [pH 7.9], 1.5 mM MgCl_2_, 10 mM KCl, 25 mM NaCl, 0.5 mM DTT, 0.2 mM PMSF), and protease inhibitors. The nuclear fraction was subsequently isolated using buffer B (25mM HEPES, 1 M NaCl, 20% glycerol, 1.5 mM MgCl_2_, 0.1 mM EDTA, 0.5 mM DTT, 0.5 mM PMSF), and protease inhibitors. Finally, the two fractions were mixed to obtain complete cell extracts. For immunoprecipitations, extracts were incubated with a rabbit monoclonal H3K27me3 antibody raised against a synthetic peptide corresponding to the N-terminus of histone H3 and containing a tri-methylated lysine 27 (Cell Signaling, #9733*)* and incubated overnight at 4 °C by end-over-end rotation. Protein A sepharose beads (GE Healthcare) were added for an additional 90 minutes at 4 °C. Beads were washed two times with IP low salt buffer followed by three washes with IP high salt buffer for 10 minutes each. Proteins were eluted off the beads by incubation at 95 °C for 5 minutes in presence of 1x SDS-PAGE loading buffer. Proteins were analyzed by gel electrophoresis followed by western blotting using a rabbit monoclonal antibody raised against the C-terminus of histone H3 (Cell Signaling #4499). For Western blots, lysates were run using 4-12% Bis tris gels (Invitrogen). 10 µg of extract was loaded per lane. H3K27me3 (Cell Signaling, #9733) and H3 (Cell Signaling, #4499) antibodies were used at a 1:1000 dilution in TBS-T. Incubation with primary antibodies was carried out overnight and with secondary antibodies (IR-dye, Thermo) for 1 h at RT. Odyssey CLx Infrared Imaging System (LI-COR Biosciences) was used for immune-signal detection.

**Histone Protein Mass Spectrometry**

For SILAC experiments, KARPAS-422 cells were seeded at 0.5x10^6^ cells/ml in RPMI-1640 without L-arginine or L-lysine, supplemented with L-arginine at 200 mg/L, L-lysine hydrochloride 12C (light) or 13C (heavy) at 40 mg/L, 10% FBS (HyClone), and antibiotics (Invitrogen). Cells were passaged 5 times before proceeding, at which point they were re-seeded as above. Cells in heavy media were treated with 0.1% DMSO, and cells in light media were treated with 0.625, 2.5, or 10 μM CPI-360 for 4 days before harvesting. 2 million DMSO-treated heavy conditioned cells were mixed with 2 million CPI-360 light conditioned cells in same tube, pelleted by centrifugation at 500 x g for 2 minutes, washed in PBS, and re-pelleted. Cells were snap frozen and stored at -80 °C until further processing. For all cell pellets to be analyzed by mass spectrometry the histone isolation was performed using EpiQuik kit (Epigentek; Farmingdale, NY) per instructions. Resultant soluble histone protein was precipitated using 20% TCA for 3 hrs on ice. After isolation the histones were propionylated (Sigma, St. Louis, MO) as previously described [1] to block free amines on the histone proteins and then treated with trypsin (Promega, Madison, WI) for overnight digestion. After digestion, resultant peptides were subjected to derivatization using phenyl isocyanate (Sigma; St. Louis, MO; submitted). Each sample was desalted via Stage Tip prior to mass spectrometric analysis. Peptides were resuspended in 0.1% formic acid and analyzed by direct online injection into a QExactive mass spectrometer (Thermo Scientific). Chromatographic separation was performed over a gradient of 0-55% acetonitrile containing 0.1% formic acid over 55 minutes on a 75 µm x 25 cm analytical column packed with MAGIC C18 3µm packing material with a flow rate of 300nl/min. Survey scans were acquired in the range of m/z 300-1750 at 70,000 resolution with poly dimethylcyclosiloxane from ambient air (m/z 445.120025) as a lock mass [2] and an AGC setting of 1e^6^. For each cycle, the 10 most intense ions were fragmented using HCD at energy of 27 with an AGC setting of 5e^4^, resolution of 17,500 and predicted charge state of z=2. The isolation width was 1.6m/z and dynamic exclusion was set to 20 sec. Masses for each form of the H3.1 27-40 peptide were identified and the location of any modifications was determined by interrogation of MS/MS scans. Heavy labeled peptides from the DMSO sample were also identified in each analysis. After all peptide species were identified in the mass spectrometric data, the AUC values were used to calculate the relative amounts of each modification state present in the sample. Additionally, AUC values for peptides containing an individual modification, were summed and graphed to determine the overall changes occurring to the modification.

**ChIP-qPCR**

Real-time PCR analysis was performed on Roche Light Cycler 480 II with the use of UPL probes (Roche Applied Science), SYBR green (Fast SYBR Green Master Mix, Applied Biosystems #438612) or SYBR Green Supermix (Bio-Rad). Each PCR reaction was performed in triplicate. Data were generated from two independent ChIP experiments. Signal intensity value for each sample was calculated from the average of the two experiments. *MYT1* and *ACTB* primers were from Active Motif (#71007 and #71023). qPCR Primer sets used in supplemental figures were:

*ABAT*: F: GCTGAACTCTTTCTCTGCCTTTA R: GCGATCACCAAGTCCTCATAA (UPL 58),

*APOL1*: F: GGTCTCAGGCTGCTCCTCTA R: CCCTCCACCTTCAGTCCAG (UPL 74),

*EPB41L1*:F:CCTTAGTTTGTGTAAGTCCTTGGAG R:AAGTGAGCAGCTGAGAACATAAGA (UPL 4),

*FBXO39*: F: TTTTGAGCCCCAAGTTCG R: ATGTGCAGAGGGCGAGAC (UPL 81),

*MPEG1*: GCCTAACAGGTAGCTATCCTAATTTTT R: GTGTGGGGTCGGGATTTT (UPL 20),

*U6-5*: F: ATACCTGGCATATGTTAGGCACTC R: TTGTCAAGACTTCTTTGCGTTTGAG (UPL 147),

*CG5276:* F: CGCCTTCGTACTCGTCCTAC R: GACCACCATTGTCCAGACTC,

Ebi: F:GACGACAATTCGCTTTAAGAATTT R: CGGAAATTTATTAGCCATAAAAACA

Dys: F:TGCCGAAAAAGGAAAAACTG R: CGTGTTGTTGGGAAGCCTAC

*Nos*: F: TGAGCCAAACTCTAACCTGCT R: GATCGCGCCTGTCTCTTC,

Mer: F: ACAGGGATCGGTTTTTCTTG R: TTGCATCTAACGCATCACATC

*Pbgs*: F: CTCCTTCAGGCGATTCAGTC R: TATCCGGTGTTCATCGTGAG,

*Slo*: F: TGAATTCGCCTTGTCTTGG R: GTCAGTACGGGCGACAAACT,

Untr3L: F:CGCCTTCGTACTCGTCCTAC R: GACCACCATTGTCCAGACTC

The resulting signals were normalized according to the ChIP-IT qPCR Analysis Kit (Active Motif #53029).

**Supporting Information References**

1. Garcia BA, Mollah S, Ueberheide BM, Busby SA, Muratore TL, Shabanowitz J et al. Chemical derivatization of histones for facilitated analysis by mass spectrometry. Nat Protoc. 2007;2(4):933-938.
2. Olsen JV, de Godoy LM, Li G, Macek B, Mortensen P, Pesch R et al. Parts per Million Mass Accuracy on an Orbitrap Mass Spectrometer via Lock Mass Injection into a C-trap. Molecular & Cellular Proteomics. 2005;4(12):2010-2021.
