# Supplementary material for: An Alternative Approach to ChIP-Seq Normalization Enables Detection of Genome-Wide Changes in Histone H3 Lysine 27 Trimethylation upon EZH2 Inhibition
Source: PLoS One. 2016 Nov 22;11(11):e0166438. doi: 10.1371/journal.pone.0166438 (PMC5119738; doi:10.1371/journal.pone.0166438)
Supplement: S1 Table — ChIP DNA was quantified using Qubit fluorometric quantitation. Total immunoprecipitated DNA mass and fold change after EZH2 inhibitor treatment are presented. (PDF) [file pone.0166438.s012.pdf]

**H3K27me3**	Total ng	DMSO/inhibitor Fold change
Karpas-422 8 days, DMSO	12.0	
Karpas-422 4 days, CPI-360	3.0	4.0
Karpas-422 8 days, CPI-360	2.4	5.0
PC9 5 days, DMSO	0.84	
PC9 5 days, GSK126	0.21	3.9
