# Supplementary material for: An Alternative Approach to ChIP-Seq Normalization Enables Detection of Genome-Wide Changes in Histone H3 Lysine 27 Trimethylation upon EZH2 Inhibition
Source: PLoS One. 2016 Nov 22;11(11):e0166438. doi: 10.1371/journal.pone.0166438 (PMC5119738; doi:10.1371/journal.pone.0166438)
Supplement: S2 Table — ChIP-seq libraries were PCR amplified for 15 cycles. ChIP-seq library yields were measured using a NanoDrop spectrophotometer. Yields within groups of treated and untreated cells were similar. (PDF) [file pone.0166438.s013.pdf]

Sample	Antibody	Library yield [ng]
KARPAS-422 DMSO 8 days	H3K27me3	90
KARPAS-422 4 days CPI360	H3K27me3	76
KARPAS-422 8 day CPI-360	H3K27me3	79
KARPAS-422 DMSO 8 days	H3K9me3	101
KARPAS-422 4 days CPI360	H3K9me3	96
KARPAS-422 8 day CPI-360	H3K9me3	99
KARPAS-422 DMSO 8 days, rep2	H3K27me3	87
KARPAS-422 4 days CPI360, rep2	H3K27me3	84
KARPAS-422 8 day CPI-360, rep2	H3K27me3	91
PC9 control cells	H3K27me3	71
PC9 EZH2 inhibitor	H3K27me3	64
PC9 control cells	H3K4me3	25
PC9 EZH2 inhibitor	H3K4me3	32
PC9 control cells, rep2	H3K27me3	67
PC9 EZH2 inhibitor, rep2	H3K27me3	60
PC9 control cells, rep2	H3K4me3	63
PC9 EZH2 inhibitor, rep2	H3K4me3	61
