# Supplementary material for: An Alternative Approach to ChIP-Seq Normalization Enables Detection of Genome-Wide Changes in Histone H3 Lysine 27 Trimethylation upon EZH2 Inhibition
Source: PLoS One. 2016 Nov 22;11(11):e0166438. doi: 10.1371/journal.pone.0166438 (PMC5119738; doi:10.1371/journal.pone.0166438)
Supplement: S3 Table — Normalization factors were calculated based on tags mapping to the entire dm3 genome (168,736,537 bp), H2Av peak regions (15,942,729 bp) or H3K27me3 peak regions (34,740,761 bp). Normalization factors were calculated from two independent ChIP-seq spike-in campaigns (replicate 1, 2). For KARPAS-422 cells, H3K27me3 ChIP-seq was carried out on cells that were treated with DMSO or 1.5 μM CPI-360 for 8 days and H3K9me3 ChIP-seq from cells treated for 4 and 8 days. For PC9 cells, H3K27me3 and H3K4me3 ChIP-seq was carried out on cells that were treated with DMSO or 1 μM GSK126 for 5 days. Numbers represent the ratio of human (top) and D. melanogaster (bottom) reads in treated versus control samples. (PDF) [file pone.0166438.s014.pdf]

Cell lines	KARPAS-422				PC9			
Antibodies	H3K27me3		H3K9me3		H3K27me3		H3K4me3	
replicate	1	2	1	2	1	2	1	2
Ratio of human reads from DMSO and EZH2 inhibitor treated samples								
All tag counts	0.342	0.278	1.228	1.051	0.231	0.196	0.836	0.869
H2Av peaks	0.333	0.236	1.187	1.091	0.238	0.286	0.853	0.872
H3K27me3 peaks	0.329	0.311	1.326	0.967	0.226	0.174	0.803	0.860
Ratio of *Drosophila* reads from DMSO and EZH2 inhibitor treated samples								
All tag counts	4.188	3.362	0.975	0.837	3.550	3.413	1.228	1.546
H2Av peaks	4.297	3.962	1.009	0.806	3.442	2.339	1.204	1.540
H3K27me3 peaks	4.343	3.010	0.903	0.910	3.632	3.842	1.279	1.562
